# Reducing tobacco supplier profits and pricing power: Modelling the impact of a tobacco price cap and tax increase on socioeconomic inequalities in England

**DOI:** 10.1016/j.socscimed.2026.119325

**Published:** 2026-08

**Authors:** Duncan Gillespie, Damon Morris, Ryan Kai Le Chen, Alan Brennan, J. Robert Branston

**Affiliations:** a Sheffield Addictions Research Group (SARG), School of Medicine and Population Health, The University of Sheffield, Sheffield, S10 2TN, United Kingdom; b School of Management and Tobacco Control Research Group, University of Bath, Bath, BA2 7AY, United Kingdom

## Abstract

**Background:**

The tobacco industry generates substantial profits from products causing significant health and societal costs. These profits enable the industry to use pricing as a flexible marketing tool. Consequently, calls exist for a scheme to cap wholesale tobacco prices and offset this with higher taxation, reducing price variation and potentially raising revenue. This study models the health and economic impact of such a scheme in England.

**Methods:**

We used the Sheffield Tobacco and Alcohol Policy Model, an individual-level microsimulation, to project tobacco consumption, spending, and health outcomes for adults in England aged 18–89 from 2025 to 2044. We investigated six scenarios for a wholesale price cap and concomitant tax rises, comparing outcomes against a business-as-usual scenario.

**Results:**

Outcomes varied with the price cap level; lower caps and higher tax rises yielded larger behavioural effects, particularly for the most disadvantaged quintile. All scenarios showed a narrower market price range, lower smoking prevalence, higher tax revenue, reduced mortality, and fewer hospital admissions. Industry revenues declined, while consumer expenditure remained largely unchanged. An immediate hard cap could generate £4.9 billion by 2029 and, by 2044, 1,636 fewer deaths, 43,987 fewer years of life lost, and 10,073 fewer hospital admissions. Sensitivity analyses show health benefits are robust, although stronger consumer responses slightly reduce tax revenue while increasing health gains.

**Conclusions:**

A tobacco wholesale price cap and tax increase scheme could raise substantial tax revenue, improve health, and reduce health inequalities, whilst limiting the scope to use price as a marketing tool.

## Highlights

• A tobacco price cap and duty rise scheme could raise substantial tax revenue.
• Raises cheap tobacco costs, cutting smoking rates and reducing health inequalities.
• Industry profits decline while pricing becomes less important as a marketing tool.

## 1 Introduction

Worldwide, tobacco use causes over 8 million annual deaths, kills up to half of its long-term users (World Health Organisation, 2023), and costs an estimated US$1.85 trillion—approximately 1.8% of global Gross Domestic Product (Vulovic, 2019). Despite world-leading tobacco control in countries like Australia, New Zealand and the United Kingdom (UK), existing regulations are widely considered insufficient. Transformative measures are required to make tobacco smoking obsolete, particularly regarding product pricing (The Lancet, 2021). The UK Government's Tobacco and Vapes Bill includes such a measure: the “smoke-free generation” policy, which will raise the legal age of tobacco sale by one year annually from 2027 (UK Government, 2026). However, as its impact will unfold over decades, there is a need for further measures aimed at people of all ages who already use tobacco products.

Taxation and other measures inducing higher prices are recognised as crucial tobacco control strategies, as they reduce affordability (World Health Organisation, 2018). The main UK tobacco products are factory-made (FM) cigarettes and hand-rolling tobacco (HRT); both are subject to specific duty per 1,000 FM cigarettes or kilogram of HRT. FM cigarettes have two further excise components: an ad-valorem duty (16.5% of retail price) and a minimum excise tax (MET)—payable if the sum of specific and ad-valorem duties falls below the MET threshold. VAT is then applied at 20% to the duty-inclusive price. Since 1993, UK tobacco tax policy has mostly employed an excise duty escalator, under which duty has risen annually by a set percentage above Retail Price Index (RPI) inflation. This has gradually reduced the affordability of tobacco (Partos et al., 2019).

To maintain profits, the tobacco industry tends to use two pricing strategies in response to tax rises. First, the industry raises wholesale prices so that retail prices increase even more than caused by the tax increase alone (known as “over-shifting” tax). Second, this over-shifting strategy is used less on cheaper products to keep them relatively affordable, whilst compensating with higher profits on other products where price plays a smaller role in consumer choice (Sheikh et al., 2023; Wilson et al., 2020). As a result, the effectiveness of taxation policies in reducing smoking prevalence depends on regulations that prevent the tobacco industry from manipulating the effects of taxation.

In 2014, the UK Government consulted on introducing a tobacco industry levy. This levy was essentially a type of excise tax, applied retrospectively and set at higher rates for companies with larger market shares, based on sales volume. Its purpose was to raise a specific amount of revenue, similar to United States Food & Drug Administration user fees and Canadian tobacco levies (Government of Canada, 2025; U.S. Food and Drug Administration, 2025). Following the consultation, the government chose not to proceed with the levy, concluding it would not raise enough additional revenue (HM Treasury, 2015). A major reason was an assumed strong consumer response to resulting tobacco price rises.

This paper explores a different policy: capping wholesale tobacco prices while substantially increasing specific excise duty (tax charged per stick or gram). The goal is to make cheaper tobacco more expensive and prevent premium retail prices from falling due to the cap (Branston and Gilmore, 2014; Gilmore et al., 2010; Scollo and Branston, 2022). This approach is gaining international support to reduce tobacco supplier profits and pricing power (FCTC measure b–iii; WHO FCTC, 2025), and in the UK under the banner of a “polluter pays levy scheme”, most recently by the All Party Parliamentary Group on Smoking and Health (APPG on Smoking & Health, 2025).

Under this scheme a government department or independent regulatory body would set the maximum wholesale price the tobacco industry could charge. This cap would be set by estimating production and import costs, adding a small, regulated profit margin comparable to other manufacturing industries. It would need periodic review, and adjustment if necessary, for inflation, production cost changes, and industry actions designed to undermine the policy. Capping wholesale, rather than retail, prices is a deliberate choice. Tobacco production is concentrated among a few large companies enjoying considerable market power and large profits (Branston, 2021), whilst distributors and retailers make very little profit (Hitchman et al., 2016). Furthermore, tobacco manufacturers have previously exploited distributors and retailers, limiting their slim margins (e.g. by producing price-marked packs) and using them as third parties to fight proposed tobacco control measures (Gilmore et al., 2015). Thus, targeting wholesale prices directly addresses the source of market power and profits, keeping the scheme simple and financially protecting distributors and retailers, giving them little ground to object. Whilst potential increased tax revenue is a significant feature, this is not primarily a revenue-raising policy. The fundamental motivation is rooted in public health. Rather than seeking a “fiscally optimal” policy to maximise government income, this approach prioritises health maximisation and reducing health inequalities by making tobacco less affordable and less profitable to sell.

This scheme offers two broad benefits. Firstly, it eliminates the industry's ability to use price as a marketing tool. Prices for products like FM cigarettes and HRT would effectively standardise across brands, reducing price differentiation. This prevents the industry from offering cheap economy brands to maintain affordability alongside expensive, profit-maximising ones, ultimately making taxation more effective by stopping pricing tactics that offset tax increases (Sheikh et al., 2023; Wilson et al., 2020). Socioeconomic benefits are expected because those favouring economy brands (e.g. young people and lower-income groups) would face higher retail prices, providing stronger signals to quit or not start. Secondly, offsetting wholesale price reductions with excise duty increases could transfer previously large industry profits to government tax revenues (Branston, 2021). A previous investigation using UK company accounts up to 2010 estimated this at approximately £500 million annually (Branston and Gilmore, 2014); however, these estimates are now dated and exclude changing market dynamics resulting from the aforementioned retail price changes.

From a government perspective, tobacco taxation involves a trade-off between public health and tax revenue. If consumers respond strongly to tax rises by reducing consumption, health improves but revenue decreases. Conversely, if consumption is broadly maintained, tax revenue increases, which may subsequently be reinvested in initiatives to reduce societal inequalities and smoking rates (Hatchard et al., 2023a, 2023b; Smith et al., 2023). This consumer response is measured by the price elasticity of demand: the percentage change in consumption resulting from a 1% price change. Empirical evidence generally indicates tobacco is “price inelastic”, meaning consumption drops proportionally less than the price increases (Nguyen et al., 2012; Pryce et al., 2023). However, estimates vary significantly; for instance, current UK Government projections assume a more reactive response, where a 1% price increase yields a greater than 1% consumption reduction (Office for Budget Responsibility, 2025). By incorporating a range of these estimates, this study acknowledges consumer response as a key uncertainty in fiscal and health forecasting (Martín-Álvarez et al., 2025), while ensuring that the core findings are grounded in the most robust contemporary evidence.

To understand how a wholesale price cap combined with tax rises could affect consumer behaviour, public health, and government revenues, this study provides an illustrative, modelled example to inform further policy development. We model the introduction of wholesale price caps on FM cigarettes and HRT in England to indicate potential UK-wide effects. Although UK tobacco duty is set centrally, this study focuses on England due to data availability; however, as England comprises nearly 85% of the UK population, the results are broadly representative (Office for National Statistics, 2024). Using an individual-based microsimulation model tracking population trends over time, we project the policy's impact on smoking prevalence, consumption amounts, consumer spending, government tax revenues, and industry net revenues (sales revenue excluding taxes). Additionally, the model assesses socio-economic differences in health outcomes, specifically mortality and hospital admissions.

## 2 Methods

We used the Sheffield Tobacco and Alcohol Policy Model (STAPM; version 2.5.1), a dynamic microsimulation tracking individuals’ tobacco use over time within the English population aged 18–89 (Morris et al., 2023, 2024). The population is socio-economically stratified by Index of Multiple Deprivation (IMD) quintiles, a small-area composite measure of socio-economic conditions (Ministry of Housing Communities and Local Government, 2019). Running in yearly steps from 1st January 2017 to 31st December 2044, the model simulates continued declines in smoking prevalence, extrapolated from recent trends, alongside all tax changes implemented prior to the 2025 policy changes investigated here. Policy effects are compared against a “business-as-usual” scenario assuming a 2% annual real-terms duty increase. This baseline excludes anticipated future UK policies, such as the “smoke-free generation” or other measures within the Tobacco and Vapes Bill (UK Government, 2026). STAPM has previously assessed tobacco and alcohol pricing policies and duty reforms in England and Scotland (Chen et al., 2026; Gillespie et al., 2025; Morris et al., 2024). Simulations were conducted in R (R Core Team, 2024). An overview of the model is presented here, with full details in Supplement A.

### 2.1 Tobacco consumption

The simulated population of 250,000 individuals was derived from pooled 2016–2018 Health Survey for England data, an annual nationally representative household survey collecting individual tobacco consumption data (NatCen Social Research, 2024). Consumption (average weekly cigarettes) is split into two types: FM and HRT. We assume a hand-rolled cigarette contains 0.5 g of tobacco (Branston et al., 2018). Smoking prevalence is projected using state transition probabilities, estimated from survey data and validated against historical trends (section 2.6 in Tobacco Advisory Group of the Royal College of Physicians, 2021). The model also tracks five alcohol products (beer, cider, wine, spirits, and alcopops), separated into on-trade (e.g. pubs, restaurants) and off-trade (e.g. supermarkets) sales channels.

### 2.2 Prices

Price distributions (expressed as price per gram) describing how purchases spread across retail prices were derived from 2006 to 2018 Living Costs and Food Survey (LCFS) data (Department for Environment Food and Rural Affairs & Office for National Statistics, 2023). As respondents record the tobacco type (FM cigarettes or HRT) and price paid but omit pack size, we imputed sizes using market research data (Wilson et al., 2021). Distributions were derived for both types across 800 modelled population subgroups and matched to individuals. Retail prices were then decomposed by reverse-calculating 20% VAT and subtracting ad-valorem (for FM) and specific duties. This residual serves as a wholesale price proxy (combined manufacturer and retailer revenue) used to apply cap adjustments.

To model a combined price cap and duty increase, we first lower any wholesale prices that exceed the cap. We then estimate new retail prices by adding the updated duty. Finally, we adjust these estimates to account for industry modification of tax pass-through behaviour; rather than passing duty changes through to retail prices uniformly, there is evidence that tobacco companies shift their net revenue to vary price increases across their product range (Wilson et al., 2020). Specifically, our model assumes heterogeneous pass-through: the price cap forces a negative or partial pass-through on premium brands (where wholesale prices must drop), while the tax increase is passed through in line with the Wilson et al. estimates, subject to the constraint imposed by the price cap. The model assumes that the industry's only response to tax changes is this price manipulation, as strict UK regulations already limit other supply-side reactions, such as new marketing strategies. The final retail price is then calculated by summing the adjusted wholesale price and the specific duty.

### 2.3 Effects on consumption and health outcomes

Changes in price affect consumption via price elasticities of demand, as estimated by Pryce and colleagues (2023). These measure the percentage change in consumption resulting from a 1% change in a product's own price (own-price elasticities) or another product's price (cross-price elasticities). Our estimates include cross-price elasticities relating tobacco prices to alcohol consumption; therefore, modelled health impacts reflect consumption changes across both product types. We apply separate elasticities for participation (consume or not) and conditional consumption (amount consumed). The basecase analysis uses only own- and cross-price elasticities that are statistically significant at the 95% level, setting non-significant values to zero (see Supplement A, p. 28). Finally, tobacco and alcohol consumption affects health across 84 ICD-10 disease categories attributable to either or both substances (see Supplement A for epidemiological details).

### 2.4 Policy scenarios

Any real-world application of a scheme of price cap regulation and compensating duty uplift would have a number of important variables to set, including the level of the cap introduced, how duty would be increased to offset reductions in wholesale prices, the extent to which the cheapest prices would increase (and hence the price range would narrow), and the speed at which the price cap/duty rises were introduced. In order to explore some of the key choices that could be taken, we modelled a variety of indicative situations. For simplicity, we assume that any given price cap would apply equally to the markets of FM and HRT products taken as a whole, as the costs of production are broadly similar and because tax accounts for the vast majority of retail prices (Wilson et al., 2020).

We firstly modelled three levels of wholesale price cap: (1) Soft cap of £0.110 per stick; (2) Moderate cap of £0.070 per stick; (3) Hard cap of £0.035 per stick, with the hard cap causing the biggest reduction in wholesale prices. The soft cap would only affect the wholesale prices of FM cigarettes. The moderate cap would affect more than half of FM cigarettes and the most expensive HRT. The hard cap would affect all FM cigarettes and more than half of the HRT market.

We modelled only one scenario for duty increases to accompany each cap level, which was based on ensuring that the average price of tobacco did not change due to the introduction of the price cap. This was done by calibrating the percentage increase in specific duty applied across all products and price points such that the average price of all tobacco stayed constant, given the wholesale price cap. Whilst other scenarios are possible, such as maximum price equivalisation so that no tobacco product becomes cheaper (either in real terms or nominal terms), the modelled average-equivalising scenario was chosen as an intermediate approach for illustrating the potential effects of the policy. The duty increases required for this approach are large, and the basecase price elasticities were estimated using LCFS data from 2006 to 2017, when tobacco tax and price increases were more modest. We linearly extrapolate the price responsiveness of consumers, assuming that the consumer response to the price changes we model remains in line with these price elasticities. Consumers may, however, change their sensitivity to price changes when the magnitudes of those changes are much larger, and there may be other behavioural responses which the model does not account for.

Finally, for all three levels of price cap, we modelled the immediate adoption of the policy from 2025, and also a 5-year phased introduction where the price cap and concomitant tax increases were introduced linearly over several years. In the phased scenario, the same price cap/duty rates are achieved in 2030 as the corresponding immediate scenarios, but by gradually lowering the cap from the highest wholesale price in 2025 and increasing specific duty in each year from its level in 2025 to 2029.

### 2.5 Modelled outcomes and sensitivity analyses

Reported outcomes include changes in tobacco use prevalence, average cigarettes smoked, and average weekly tobacco expenditure per person who smokes. Industry net revenues and government tax revenues (duty receipts plus VAT) are reported cumulatively for a 5-year period (2025–2029), aligning with the 5-year economic forecasting undertaken by the UK Government when setting fiscal policy. Modelled health outcomes included annual all-cause deaths, total years of life lost, hospital admissions, and the associated National Health Service (NHS) treatment costs. These health outcomes were reported cumulatively over 20 years to allow for the lag between consumption changes and disease outcomes, and are presented separately by IMD quintile. All monetary outcomes are undiscounted and reported in 2025 prices. Uncertainty could not be quantified probabilistically due to data and computational limitations. Instead, we undertook structural sensitivity analyses (see Supplement B) of the price elasticities used by investigating three alternative assumptions: (1) using all cross-price elasticity estimates (i.e. including non-significant estimates that were set to zero in the basecase); (2) excluding the statistically significant cross-price effects between tobacco and alcohol products, thereby testing the influence of cross-effects to alcohol on the basecase findings; and (3) an adjustment to the price elasticities which reproduces the same impact on total consumption of FM cigarettes and HRT as using elasticities estimated by HM Revenue & Customs (HMRC), which are used in their tobacco duty forecast model (Office for Budget Responsibility, 2025). See Supplement A (pp. 40–42) for details of the price elasticity adjustment.

## 3 Results

Table 1 describes the modelled baseline situation in 2025, prior to the introduction of the price cap and concomitant tax rise, for tobacco consumption, expenditure, industry and government revenue, and the health consequences of smoking. Among people living in the most deprived quintile of areas, 24.6% smoke tobacco, with each person who smokes consuming an average of 82.1 cigarettes per week (of which 44% are hand-rolled), spending an average of £43 per week on cigarettes. Per 100,000 people living in these most deprived areas, tobacco use was estimated to account for 165 deaths in the year (14,347 deaths total) and 513 hospital admissions (44,735 total). This is compared to 48 deaths (4,083 total) and 154 hospital admissions (13,064 total) among people living in the least deprived areas.

**Table 1:** Baseline population, consumption, economic and health outcomes by Index of Multiple Deprivation quintile (IMDQ) in 2025, before the wholesale price cap and tax increase are applied.

	IMDQ-1 (least deprived)	IMDQ-2	IMDQ-3	IMDQ-4	IMDQ-5 (most deprived)	Population
**Population (millions)**						
Population	8.47	8.61	8.79	9.14	8.72	43.74
Smoking prevalence	7.44%	11.17%	13.53%	17.61%	24.64%	14.95%
Number of people who smoke	0.63	0.96	1.19	1.61	2.15	6.54
**Tobacco consumption (average cigarettes sticks per person who smokes per week)**						
Cigarette sticks consumed per week	71.53	75.87	71.58	79.20	82.08	77.53
Percentage of total consumption that is hand-rolling tobacco	27%	40%	39%	50%	44%	43%
**Tobacco prices and spending (£ per person who smokes)**						
Price per stick (All tobacco)	0.59	0.54	0.54	0.50	0.52	0.53
Price per stick (Cigarettes)	0.69	0.69	0.69	0.68	0.68	0.69
Price per stick (Hand-rolled tobacco)	0.32	0.32	0.32	0.32	0.32	0.32
Spend per week	42.33	40.97	38.86	39.58	42.96	41.03
**Annual tobacco industry revenue and government tax revenue (£ billion)**						
Industry revenue	0.26	0.37	0.44	0.59	0.85	2.50
Tobacco duty + VAT	1.13	1.68	1.97	2.72	3.95	11.45
**Tobacco attributable health outcomes**						
Deaths	4,083	5,458	6,984	9,753	14,347	40,625
Admissions	13,064	17,689	22,838	31,543	44,735	129,870
**Tobacco attributable health outcomes (rates per 100,000 population)**						
Standardised deaths	48	63	79	107	165	93
Standardised admissions	154	205	260	345	513	297

**Fig. 1:**
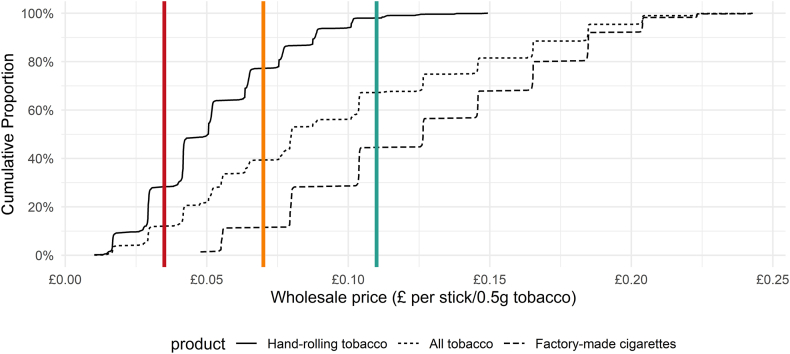
How the three price caps affect the distribution of wholesale prices for FM cigarettes and HRT, separately and combined. The three vertical lines (from left to right) indicate the hard, moderate and soft caps.

The modelling estimated that a hard cap of £0.035 per stick would require a 23.6% duty rise, whilst the moderate (£0.070) and soft caps (£0.110) would require smaller 13.9% and 6.6% duty rises to maintain average prices at their 2025 levels. Fig. 1 illustrates how the three price caps affect the distribution of wholesale prices for FM cigarettes and HRT, separately and combined. The soft cap (£0.110) affects the upper half of the wholesale price distribution for FM cigarettes, but only a small share of the most expensive HRT products. The moderate cap (£0.070) affects almost all FM cigarettes and approximately the top 20% of HRT products. The hard cap (£0.035) would lower wholesale prices for all FM cigarettes and the top 70% of HRT products. When considering the combined effect on FM cigarettes and HRT together, the soft, moderate, and hard caps affect approximately the top 30%, 60%, and 90% of wholesale prices, respectively.

**Fig. 2:**
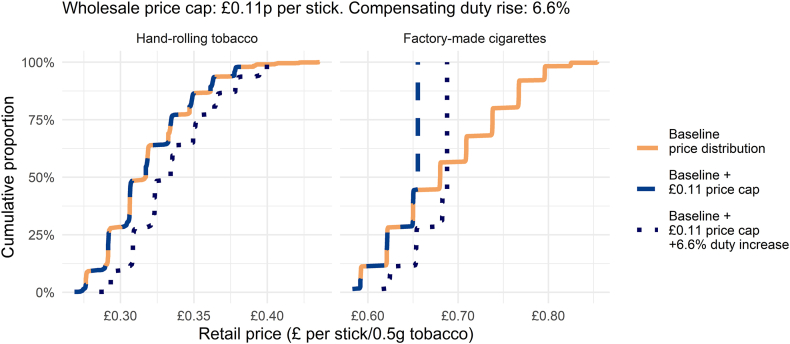
The effects of the soft (£0.11) wholesale price cap and the compensating 6.6% increase in excise duty on retail price distributions for FM cigarettes and HRT. Each panel shows three lines that indicate the baseline retail price distribution, how the price cap by itself affects retail prices, and then the effect of the additional duty increase.

Fig. 2 illustrates the effects of the soft (£0.110) wholesale price cap and the compensating 6.6% increase in excise duty on retail price distributions for FM cigarettes and HRT. Under the baseline scenario, FM cigarette prices range from just under £0.60 to around £0.80 per stick, while HRT prices range from approximately £0.25 to £0.40 per stick. Introducing the soft price cap reduces the retail prices of the most expensive FM cigarettes, narrowing the overall price distribution. In contrast, HRT prices remain largely unchanged, as the soft cap affects only a small proportion of HRT products at the wholesale level. Applying the 6.6% duty increase then shifts the retail price distributions for both products to the right. This clearly raises the average price paid for HRT. For FM cigarettes, prices do rise compared to a “price cap only” scenario. However, because of the larger cost-lowering effect of the cap on the more expensive products, some products remain cheaper than they were originally at baseline.

**Fig. 3:**
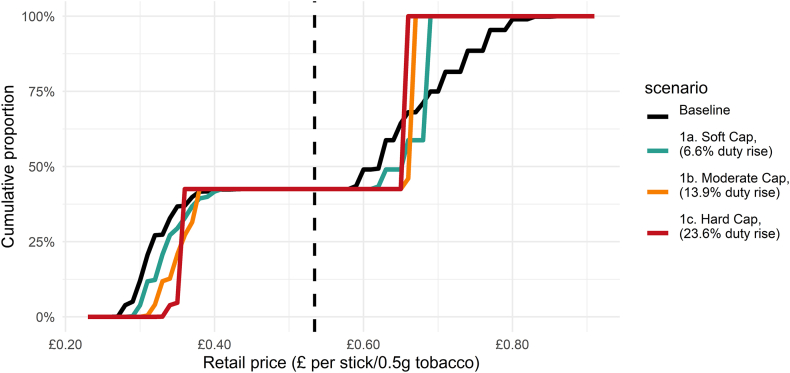
Impact of a wholesale price cap and immediate average retail price-equivalising duty increases on the price distribution for all-tobacco. The vertical dashed line represents the average retail price of all tobacco products at baseline.

Fig. 3 presents the combined retail price distribution for FM cigarettes and HRT. The distribution displays two distinct peaks, reflecting the clear separation in prices between the two products: the lower-priced peak corresponds entirely to HRT, while the higher-priced peak corresponds entirely to FM cigarettes. As a result, HRT dominates the lower end of the retail price distribution, and FM cigarettes dominate the higher end. The figure shows how the three price cap policies narrow the range of retail prices available in the tobacco market, both within each product category and across the market as a whole. The wholesale price cap reduces the prices of the most expensive tobacco products, compressing the upper end of the distribution, while the compensating duty increase raises prices at the lower end. Together, these effects compress retail prices around the overall average tobacco price. The combined result is a compression of the price distributions for both FM cigarettes and HRT, and a narrowing of the price gap between the two products.

Table 2 shows the modelled estimates for changes in smoking prevalence by 2030 as a result of the different wholesale price cap and equivalising duty rise scenarios. The results show that there would be fewer people who smoke, more government tax revenue, less revenue to the tobacco industry, and fewer tobacco-related health harms. In 2030, the largest reduction in smoking prevalence (0.08%, or 35,437 fewer people who smoke) comes from the hard £0.035 wholesale price cap and 23.6% duty rise immediately applied. This effect would be largest at 0.13% (11,853 fewer people who smoke) in the most deprived population subgroup. In all scenarios, average weekly tobacco spending remains broadly unchanged. Tobacco industry retail revenue from tobacco decreases by up to £7.8 billion over 5 years. In contrast, tax revenue from tobacco increases by £1.2 billion with the soft (£0.110) cap and £4.9 billion with the hard (£0.035) cap. We estimated a reduction of 1,636 deaths in the hard cap scenario, alongside 43,987 fewer years of life lost to death and 10,073 fewer hospital admissions. The phased scenarios offer smaller returns over 5 years but might allow people to adjust gradually to the changes in market prices.

**Table 2:** Impact of simultaneous price cap and compensating duty rise policies on smoking prevalence, spending, revenues, and health outcomes.

		Intervention arms (absolute difference from Control)					
	Control	Soft (immediate)	Moderate (immediate)	Hard (immediate)	Soft (phased)	Moderate (phased)	Hard (phased)
**Smoking prevalence in 2025 (% of population)**							
Population	14.95	−0.11	−0.20	−0.29	0.01	−0.02	−0.05
**Smoking prevalence in 2030 (% of population)**							
Population	13.00	−0.03	−0.05	−0.08	−0.02	−0.05	−0.11
IMDQ-1 (least deprived)	6.19	0.00	0.00	−0.02	−0.01	−0.01	−0.04
IMDQ-2	9.81	−0.02	−0.04	−0.07	−0.03	−0.05	−0.07
IMDQ-3	11.11	−0.02	−0.04	−0.06	0.00	−0.03	−0.07
IMDQ-4	15.10	−0.05	−0.08	−0.12	−0.02	−0.06	−0.13
IMDQ-5 (most deprived)	22.08	−0.05	−0.08	−0.13	−0.04	−0.10	−0.21
**Average tobacco consumption (cigarettes per person who smokes per week)**							
2025	77.53	−1.50	−2.80	−4.27	0.27	−0.13	−0.68
2030	76.69	−0.72	−1.16	−2.35	−0.26	−1.13	−1.78
**Average spending on tobacco (£ per person who smokes per week)**							
2025	41.03	−0.60	−1.18	−1.87	−0.23	−0.24	−0.24
2030	43.41	0.28	0.17	−0.03	−0.26	−0.23	−0.24
**5-year cumulative impact (2025**–**2029) on tobacco industry and government tax revenues (£ billion)**							
Tobacco industry revenue	11.9	−2.0	−4.5	−7.8	−1.1	−2.0	−3.0
Tobacco duty + VAT	56.0	1.2	2.8	4.9	0.4	1.1	2.1
**20-year cumulative impact (2025**–**2044) on deaths, years of life lost, admissions and admission costs**							
Deaths	9,590,708	−802	−732	−1,636	220	−856	−1,510
Years of life lost	142,023,556	−18,875	−33,963	−43,987	1,445	−23,663	−36,381
Hospital admissions	40,662,710	−5,174	−8,011	−10,073	−3,443	−7,056	−8,839
NHS admissions costs (£ million)	74,799	−10	−15	−19	−8	−14	−17

IMDQ = Index of Multiple Deprivation Quintile.

The “phased” scenarios achieve the same duty rates in 2030 as the corresponding “immediate” scenarios but by increasing specific duty by the same percentage in each year over 2025–2029. The price cap for these scenarios is introduced gradually over 2025–2029.

In sensitivity analysis S1, including all cross-price elasticities led to a greater estimated reduction in smoking prevalence and a corresponding decrease in projected tobacco tax revenue (Table 3). This effect was mainly due to the inclusion of the non-significant cross-price elasticities between FM cigarettes and HRT, which were estimated to behave as complements: when consumption of one falls, so does the other. Sensitivity analysis S2 showed that the statistically significant cross-price elasticities between tobacco and alcohol had minimal impact. The slight decrease in effect when these were set to zero stemmed from the loss of an alcohol-related mortality reduction seen in the basecase, leading to less tobacco tax revenue due to shorter lifespans among people who both smoke and drink. Sensitivity analysis S3 shows that calibrating the basecase tobacco price elasticities to those implied by HMRC estimates leads to substantially larger estimated reductions in smoking prevalence and tobacco-attributable deaths. This reflects the fact that the HMRC elasticities assume a considerably stronger consumption response to a given price change than those used in the basecase. When these stronger responses are applied in the model, the resulting reduction in consumption is sufficient to outweigh the increase in tax per unit sold, leading to estimated decreases in tobacco duty and VAT receipts over the five-year period. These revenue reductions range from £1.1 billion to £1.7 billion, in contrast to the revenue increases observed in the basecase and other sensitivity analyses. This outcome reflects the assumption that the strong consumption response implied by HMRC estimates is realised over the modelled period, resulting in a rapid adjustment in consumption. However, it is also possible that the assumed consumption response could happen more gradually over a longer period.

**Table 3:** Summary findings of sensitivity analyses of the basecase results for the three levels of wholesale price cap when immediately introduced to alternative price elasticity of demand assumptions.

Policy scenario	Basecase: Pryce et al. using statistically significant own and cross-price elasticities for tobacco and alcohol	S1: Use all cross-price elasticities including non-statistically significant ones	S2: Exclude statistically significant cross-price elasticities between tobacco and alcohol	S3: Pryce et al. calibrated to HMRC elasticities
**Difference in 2030 prevalence of smoking for Intervention vs. Control**				
(a) Soft cap, immediate implementation	−0.11	−0.2	−0.1	−0.38
(b) Moderate cap, immediate implementation	−0.2	−0.38	−0.19	−0.73
(c) Hard cap, immediate implementation	−0.29	−0.57	−0.29	−1.1
**Difference in Government Tobacco Duty + VAT 2025**–**2029 for Intervention vs. Control (£bn)**				
(a) Soft cap, immediate implementation	1.2	0.2	0.6	−1.1
(b) Moderate cap, immediate implementation	2.8	0.9	2.4	−1.5
(c) Hard cap, immediate implementation	4.9	2.2	4.5	−1.7
**Difference in 20-year (2025**–**2044) deaths for Intervention vs. Control**				
(a) Soft cap, immediate implementation	−802	522	42	−3,616
(b) Moderate cap, immediate implementation	−732	−1,751	−519	−3,600
(c) Hard cap, immediate implementation	−1,636	−1,625	−863	−6,417

## 4 Discussion

This is the first study to estimate the health and economic impact of a tobacco wholesale price cap combined with a tax increase. Reducing price variance constrains the industry's ability to offer budget entry points for price-sensitive smokers. Our basecase shows this scheme could raise over £1.1 billion in tax revenue over five years, reduce smoking prevalence, improve health, and reduce health inequalities. Stronger effects are possible with lower caps and larger, faster tax rises—for example, a hard cap with a 23.6% duty rise could raise £4.9 billion. Importantly, this comes from the tobacco industry, not consumers. Sensitivity analyses testing different consumer response assumptions show the modelled prevalence and health benefits remain robust. However, assuming stronger consumer responses, as in current UK Government estimates (Office for Budget Responsibility, 2025), slightly reduces tax revenue because consumption drops significantly—a phenomenon described by the Laffer Curve (Martín-Álvarez et al., 2025). By regulating industry margins, the policy shifts the Laffer curve itself rather than merely moving the market along it. Restricting over-shifting and narrowing price dispersion allows the government to reclaim fiscal space previously exploited by the industry to maintain consumption. Crucially, this stronger response delivers greater health benefits, illustrating an important long-term pattern: as smoking declines, tax revenues fall, yet societal benefits grow through improved health and reduced inequalities.

Conceptually, this study prioritises health and welfare over fiscal optimisation. While taxation policy often balances maximum revenue with minimal disruption, the “optimal” outcome in tobacco control is reducing consumption toward zero. Combining a wholesale price cap with a tax rise could allow the government to pursue health goals—by removing the industry's ability to maintain affordable product ranges—without lost tax revenue. By redirecting industry profits into the public purse, the policy ensures the remaining tobacco market serves the public good rather than corporate interests, even as the ultimate goal remains eradicating smoking-related harm.

While previous UK modelling explored pricing policies like tobacco minimum pricing (Gillespie et al., 2025) and steeper tax increases on HRT than FM cigarettes (Chen et al., 2026), this is the first to assess combining taxation with a wholesale price cap. Unlike minimum pricing, which boosts industry profits, this approach raises retail prices through substantial specific excise duty increases while directing revenue to the government. By restricting the industry's ability to over-shift taxes (raising prices beyond tax increases to boost profits), the government effectively reclaims “fiscal space”. This limits the industry's power to manipulate the tax system, ensuring price increases serve public health and the public purse rather than corporate interests. Captured industry profits could then be invested in health and economic initiatives, including further reducing smoking rates. Future measures, such as the smoke-free generation policy and additional funding for stop smoking services, may accelerate smoking declines but must sit within a broader strategy that continues raising the price of the cheapest tobacco and limiting price-based marketing.

To illustrate the potential effects of the wholesale price cap and duty rise scheme, we modelled simplified scenarios. Specifically, we calibrated the accompanying duty increase to keep the average retail tobacco price unchanged following the cap's introduction. This average-equivalisation approach provides a conservative, transparent baseline demonstrating the policy's core mechanism—shifting the price distribution and reducing excess manufacturer profits—without imposing the very large tax increases required by alternative calibration rules. Although we do not explicitly model variation in proprietary industry costs (e.g. unit production or distribution margins), these effectively act as a constant floor in our simulations. Because production costs represent a very small share of retail prices, reasonable variation does not alter the direction of the identified revenue transfer.

For real-world implementation, policymakers face several important design choices. First, price caps and duty increases could be defined differently for FM cigarettes and HRT. Second, while we modelled a simple linearly phased implementation, alternatives could require specific annual price conditions. Third, alternative duty calibration targets exist. For example, ensuring no tobacco product becomes cheaper, although this would require substantially larger duty rises, particularly given outliers with high wholesale prices. Under our basecase elasticity assumptions, this approach generates larger prevalence reductions and tax revenues; assuming stronger consumer responses, it yields lower revenues but even greater health gains. These design choices also interact: a phased implementation could be used to balance upward pressure on the cheapest prices while avoiding nominal price reductions for the most expensive products. Finally, our analysis focuses on capturing revenue at the point of sale through the combined wholesale price cap and excise duty mechanism.

A key strength of this study is its use of the STAPM individual-level simulation model, which precisely estimates the impact of tobacco pricing policies on consumer prices and future smoking prevalence (Morris et al., 2023). By incorporating newly estimated UK price elasticities for tobacco (separating FM and HRT) and alcohol (Pryce et al., 2023), the model captures changes in both smoking participation and consumption levels, simulating both cessation and reduced use. The centrality of elasticity assumptions to our findings reflects a long-standing debate in tobacco economics. Using Pryce et al. (2023) provides a contemporary, UK-specific empirical grounding that accounts for product substitution between FM cigarettes and HRT. These estimates align with the “mid-range” of international evidence for high-income countries, where tobacco price elasticities typically fall between −0.3 and −0.7 (U.S. National Cancer Institute & World Health Organization, 2016; International Agency for Research on Cancer, 2011). By contrast, our sensitivity analysis using the UK Government's estimates (Office for Budget Responsibility, 2025) represents a more “elastic” scenario. Engaging with this range ensures our results are robust to different assumptions about consumer responses to price shocks. While higher elasticity yields more conservative revenue projections, it also produces even more pronounced public health and health inequality reduction benefits, suggesting our basecase health gains could be conservative. Finally, our economic analysis likely underestimates total societal savings by excluding broader health costs and productivity gains (ASH, 2024; Morris et al., 2026), alongside the potential benefits of reinvesting increased tax revenue into health initiatives like smoking cessation support.

A key limitation of this study is the uncertainty surrounding consumer responses to tobacco price increases—specifically, whether higher prices lead people to quit smoking or switch products, and how broader economic factors, such as the cost of living or unemployment, influence these decisions. Evidence from Pryce et al. (2023) suggests higher prices reduce consumption of both FM cigarettes and HRT, although wide confidence intervals mean limited substitution cannot be ruled out. Our sensitivity analysis incorporating cross-price elasticities resulted in larger smoking prevalence reductions but reduced the maximum projected revenue gain from £4.9 billion to £2.2 billion over five years (Table 3), highlighting how sensitive revenue estimates are to consumer behaviour assumptions. Three additional limitations apply. First, the model excludes substitution to other nicotine products like cigarillos, heated tobacco, or e-cigarettes. While switching could be substantial following large tax rises, insufficient UK-specific evidence on cross-price elasticities and long-term health risks currently precludes robust quantitative modelling without relying on arbitrary assumptions (Squires et al., 2025). Second, our price elasticities are estimated from a period of relatively small, incremental tax increases. Applying these to the large modelled tax rises assumes consumer responses scale linearly with price changes. In practice, responses to large shocks may differ; if “tipping points” trigger non-linear surges in cessation, our health gain estimates may actually be conservative. Third, we do not explicitly model illicit tobacco or cross-border purchasing. While higher prices and reduced budget availability might incentivise seeking non-duty paid sources, the extent depends heavily on enforcement. Furthermore, the UK's island geography limits the convenience of legal cross-border purchasing compared to continental Europe. Quantitatively assessing illicit responses requires modelling enforcement and supply-side dynamics, for which there is currently no established empirical framework. Together, these limitations suggest that while tax revenue estimates are uncertain, the direction and magnitude of health and inequality impacts remain robust. Future work integrating nicotine alternatives and illicit markets will be important as the evidence develops.

Targeting domestic wholesale prices provides the most reliable basis for evaluating fiscal transfers and public health impacts, given multinational tobacco companies' scope to shift corporate tax liabilities via transfer pricing. While addressing these complexities requires detailed policy design, they do not detract from our central finding: combining a wholesale price cap with a duty increase can improve health and reduce inequalities by reshaping the price distribution and redirecting excess industry profits into public revenue. As no country has yet implemented this combined scheme, legal challenges from the tobacco industry would be expected. Similar challenges met other ground-breaking measures (Zhou et al., 2019), such as Australia's introduction of standardised packaging (Jarman, 2013). Though costly and time-consuming, governments have a strong record of ultimately prevailing, after which other countries routinely adopt the measures. Furthermore, the UK has a long track record of using price cap regulation in markets where firms enjoy market power, particularly in public utilities like water, gas, and electricity (Parker, 1997). This precedent offers a strong foundation for building a solid legal basis to enact such a scheme.

This case study applies the proposed wholesale price cap and duty rise scheme to England, a country with relatively high tobacco taxes by international standards. While policymakers in other high-tax countries can use these results to anticipate market changes, the findings also hold relevance globally. The relationship between national wealth and smoking rates is non-linear; as economies develop, tobacco consumption initially rises before eventually declining as public health awareness grows (Del Arco-Osuna et al., 2025). As developing nations progress along this curve and increasingly rely on taxation to drive down smoking rates, they are likely to encounter the same industry pricing strategies currently seen in the UK. A wholesale price cap therefore provides governments with a proactive tool to maintain a stable, equitable tax system as these long-term economic and behavioural shifts occur.

Our modelling assumes that industry responses to tobacco tax rises operate primarily through changing net revenue to manipulate retail prices, and does not explicitly incorporate alternative strategic behaviours such as changes to pack size, product composition, promotions, or supply-chain restructuring. In the UK context, many of these responses are already constrained by existing regulations, including standardised packaging, minimum pack sizes, and comprehensive bans on tobacco promotion. Moreover, because the proposed policy targets the wholesale price at the point of domestic market entry, scope for circumvention through cross-border supply-chain adjustments is limited. To the extent that industry behaviour did evolve in unanticipated ways, this would most likely reduce projected revenue gains or delay health impacts, rather than reverse the direction of effects. Importantly, the iterative design of the scheme would allow the cap and accompanying duty to be reviewed and adjusted over time, providing a safeguard against strategic industry adaptation. In addition, this policy approach is likely to be easiest to implement in countries with a history of using tobacco taxation as a central component of tobacco control, as this is more likely to imply the presence of the institutional capacity required to introduce, monitor, and periodically revise a wholesale tobacco price cap alongside accompanying tax increases.

Several related policies warrant consideration alongside this scheme. Tax increases should be coupled with robust enforcement against illicit tobacco supply (Davies et al., 2024; Jackson et al., 2024), although recent UK evidence suggests price increases have not historically driven illicit trade (Partos et al., 2020). Given the tobacco price rises under a price cap and duty rise scheme, enforcement action is essential to ensure demand for relatively cheap tobacco is met by cessation support rather than illicit alternatives. The scheme must also be paired with sustained investment in smoking cessation services, particularly for populations facing greater difficulty quitting who would otherwise pay more to smoke. A core rationale for this policy is its potential to raise substantial government funds, which could be directly reinvested into such cessation support.

In conclusion, these results are particularly relevant for nations in the “endgame” phase of tobacco control, where traditional excise increases face diminishing returns due to industry pricing strategies. In such contexts, a combined price cap and tax increase could raise government revenue, improve health, and reduce inequalities, while simultaneously reducing tobacco industry profitability. More broadly, the findings illustrate how price regulation can complement excise taxation by shifting excess manufacturer profits into public revenue, narrowing price distributions, and strengthening the health impact of existing tax policies. However, supplementary measures are essential to maximise these health and equity gains, including reducing the illicit tobacco supply and supporting people who rely on relatively cheap tobacco to quit.

## Open access statement

For the purpose of open access, the author has applied a CC BY public copyright licence to any Author Accepted Manuscript version arising from this submission.

## Ethics statement

Ethical approval was not required for this study as it involved the secondary analysis of de-identified, aggregated/anonymized data for modelling purposes. Access to and use of the datasets used to produce the model inputs was reviewed by the Research Ethics Committee of the School of Medicine and Population Health at The University of Sheffield. Specifically, use of English hospital episode statistics data (ethics ref: 039767), counts of deaths and population sizes (ethics ref: 023092), analysis of the Living Costs and Food Survey (ethics ref: 006733), and analysis of the Health Survey for England (ethics ref: 043558). All research was performed in accordance with relevant institutional and national guidelines for the use of secondary data.

## CRediT authorship contribution statement

**Duncan Gillespie:** Conceptualization, Data curation, Formal analysis, Funding acquisition, Methodology, Project administration, Validation, Writing – original draft, Writing – review & editing. **Damon Morris:** Conceptualization, Data curation, Formal analysis, Methodology, Project administration, Validation, Visualization, Writing – original draft, Writing – review & editing. **Ryan Kai Le Chen:** Data curation, Formal analysis, Writing – review & editing. **Alan Brennan:** Conceptualization, Funding acquisition, Methodology, Project administration, Supervision, Writing – review & editing. **J. Robert Branston:** Conceptualization, Funding acquisition, Methodology, Validation, Writing – original draft, Writing – review & editing.

## Declaration of competing interest

JRB owns 10 shares in Imperial Brands for research purposes. The shares were a gift from a public health campaigner and are not held for financial gain or benefit. All dividends received are donated to health related charities, and proceeds from any future share sale or takeover will be similarly donated. All other authors declare no conflicts of interest.

## Data Availability

All data inputs to the modelling are publicly available or available on application to the data owners and are detailed, along with their sources, in Supplement A.
