# Supplementary material for: Reducing tobacco supplier profits and pricing power: Modelling the impact of a tobacco price cap and tax increase on socioeconomic inequalities in England
Source: Soc Sci Med. 2026 Aug;402:119325. doi: 10.1016/j.socscimed.2026.119325 (PMC13249003; doi:10.1016/j.socscimed.2026.119325)
Supplement: Multimedia component 1 [file mmc1.pdf]

Supplement A. Supplementary model methods description

Table of contents

1. Introduction.....2
   1.1 Joint Tobacco and Alcohol Policy Modelling.....3
   1.2 The STAPM Platform.....3
   1.3 Model overview.....3
   1.4 Description of the model structure.....4
   1.5 Initialising the model.....5
      1.5.1 Initial population.....5
      1.5.2 Random number seeds.....5
2. Data.....5
   2.1 Population size data.....5
   2.2 Health Survey for England.....6
      2.2.1 Drinking data.....6
      2.2.2 Smoking data.....6
   2.3 The Living Costs and Food Survey.....7
   2.4 Mortality data.....7
   2.5 Hospitalisations data.....11
3. Microsimulation of tobacco consumption dynamics.....11
   3.1 Smoking participation.....11
      3.1.1 Period dynamics of smoking.....13
      3.1.2 Dynamic changes to consumption of tobacco for smokers.....14
      3.1.3 Estimating change to the preference between factory-made and handrolled cigarettes.....15
4. Modelling the relationship between consumption and harms.....16
   4.1 Relative risks of disease.....16
      4.1.1 Chronic diseases wholly attributable to long-term average levels of alcohol consumption.....17
      4.1.2 Diseases partially attributable to acute alcohol consumption.....17
      4.1.3 Diseases wholly attributable to acute alcohol consumption.....18
      4.1.4 Lag times from changes in alcohol consumption to changes in chronic disease risk.....19
      4.1.5 Declines in risk over time after quitting smoking.....19

4.2 Calculating population attributable fractions to estimate the total avoidable burden of mortality and morbidity due to tobacco and alcohol in a single year 20
4.3 Linking behaviour change to change in mortality and morbidity between years in the microsimulation........ 21
4.4 Mortality analysis........ 21
4.5 Morbidity analysis........ 23
5. Modelling the relationship between price and consumption........ 23
5.1 Overview of policy to price modelling........ 24
5.2 Price distributions........ 25
5.3 Price elasticities of demand........ 26
5.4 Base Case and Sensitivity Analysis........ 27
5.5 Updating consumption........ 28
5.5.1 Adjusting participation........ 28
5.5.2 Adjusting conditional consumption........ 29
6. Modelling tax policy to price........ 29
6.1 Illustrative policies........ 31
6.2 Calculate expected price........ 32
6.3 Calculating the intervention effect........ 33
6.3.1 Average price change faced by consumers........ 33
6.3.2 Calculation of net treatment effects........ 34
6.3.3 Counterfactual adjustment........ 34
6.4 Minimum excise tax........ 35
6.4.1 Relaxing the binding MET assumption........ 36
7. Modelling wholesale price cap policy to price........ 38
8. Calculating economic outcomes........ 39
8.1 Calculating total spending, tax receipts, and industry revenue........ 39
8.2 Upshifting........ 39
9. Sensitivity Analysis........ 40
References........ 43

1. Introduction

This methodological appendix provides a detailed technical explanation of the Sheffield Tobacco and Alcohol Policy Modelling (STAPM). STAPM was developed in R and R studio. The

data and code in the STAPM platform can be used to construct different types of models. The Tobacco and Alcohol Tax and Price Intervention Simulation Model (TAX-sim) which is used in this article is a model within the STAPM framework which simulates effects of tax and pricing policies on tobacco and/or alcohol consumption and computes consequent health and economic outcomes.

1.1 Joint Tobacco and Alcohol Policy Modelling

TAX-sim models the dynamics of both alcohol and tobacco and integrates their disease epidemiology. This means that a longer disease list of 84 tobacco and/or alcohol related diseases are considered. While the present article is tobacco focused, the dynamics of the alcohol component of the model are still important as they drive mortality and morbidity outcomes.

1.2 The STAPM Platform

The STAPM platform is a basis for three different models which are used in the appraisal of tobacco and alcohol tax and price interventions:

- The Sheffield Alcohol Policy Model in R (SAPM-R)
- The Sheffield Tobacco Policy Model (STPM)
- Tobacco and Alcohol Tax and Price Intervention Simulation Model (TAX-sim)

The SAPM-R and STPM models respectively model the dynamics of alcohol and tobacco consumption, using an individual-level micro-simulation approach. The TAX-sim model considers both alcohol and tobacco consumption in the same model. It does this to enable fair comparisons between alcohol and tobacco policy, and it uses the underlying components of the SAPM-R and STPM models to inform the modelling of price and tax intervention effects.

The material in this appendix draws primarily on the STPM and TAX-sim documents. Details of the alcohol modelling are not discussed in this supplementary appendix, as the modelling in the present analysis does not contain any alcohol specific policy analysis or outcomes.

This supplementary appendix is produced from existing technical documentation which details the input data used and methodologies underpinning these three models. The key documents which this appendix draws on are:

- TAX-sim technical report[1]
- STPM technical report[2]

For further details on how the alcohol dynamics in the model work, see:

- SAPM-R technical report[3]

1.3 Model overview

The STAPM method allows the simulation of entire populations at an individual level, tracking individual transitions in tobacco and alcohol consumption as they age. Individuals are indexed by age (single years, from 18 years to 89 years), sex and socio-economic conditions in terms of quintiles of the Index of Multiple Deprivation (IMDQ). The model is based on a synthetic population of representative individuals drawn from survey data. Simulated individuals are stratified into 800 population subgroups defined by age, sex, deprivation status measured by Index of Multiple Deprivation (IMD) quintile, five categories of tobacco consumption

(including non-smoker), and three drinker categories (moderate [no more than 14 units/week], hazardous [>14 units, but no more than 35 units for women and 50 units for men] and harmful [>35/50 units/week for women/men respectively]), plus abstainers. This detailed stratification allows population heterogeneity in purchasing preferences, prices paid, and risks of harm, which previous research suggests substantially affects the outcomes of alcohol pricing policies.

1.4 Description of the model structure

An overview of the model structure for the present analysis:

- Population: **England**
- Age range: **18-89 years**
- Simulated population size: **250,000**
- Index year (the year in which the model is initiated): **2017**
- Policy effect year (the year in which the interventions are applied): **2025**
- Time step: The simulation "clock" ticks forward one year at a time, i.e., y moves on to y + 1, and during that time the individual has his or her birthday and ticks onwards in age from a to a + 1.
- Time horizon (the year up to which the model is run): **2044**
- Price year (the year that real terms prices correspond to): **2025**

The 'index year' of the model is the year in which the model is initialised. The 'synthetic population' in the index year is the population sample of individuals aged 18-89 that provides the starting distributions of tobacco and alcohol consumption according to age, sex and English index of multiple deprivation quintiles (IMDQ). A high-level schematic of the TAX-sim model used in this analysis is provided in the following figure.

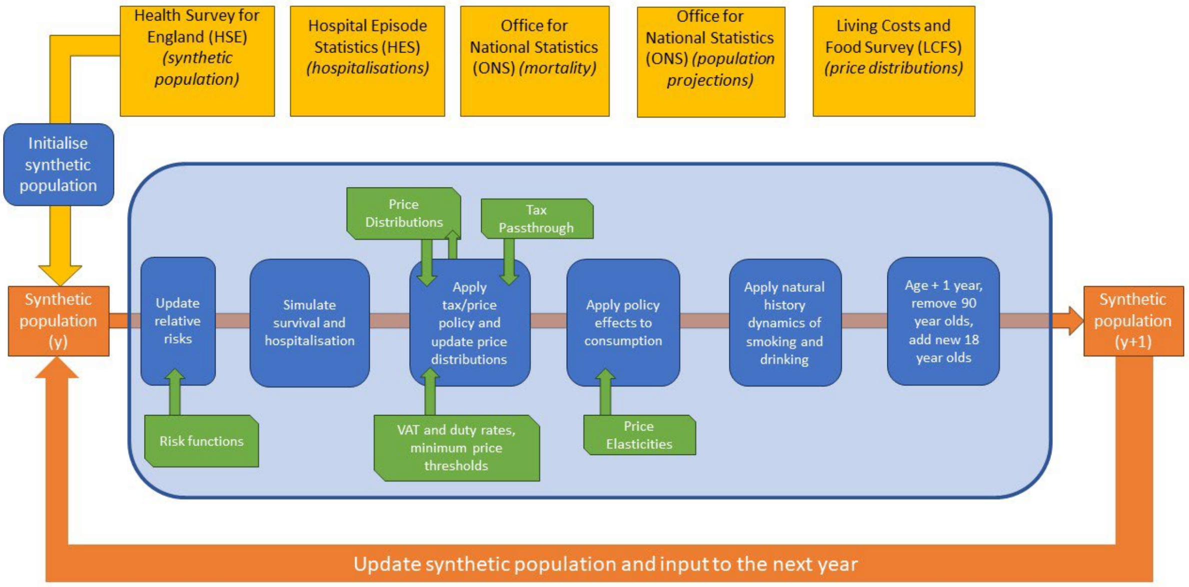

Tobacco and Alcohol Tax and Price Intervention Simulation Model (TAX-sim) for England

TAX-sim model schematic, parameters, and data sources

The synthetic population in the index year is a weighted bootstrap sample with replacement of 250,000 individuals from the Health Survey for England years 2016 to 2018. The variables retained in the synthetic population are single year of age, sex, and IMDQ. Individuals are then put into the categories of level of smoking and levels of drinking. To minimise noise in the Monte Carlo simulation between the control and intervention arms, individuals are assigned a set of random seeds that minimise the random differences between the two arms.

1.5 Initialising the model

1.5.1 Initial population

The "index year" of the model is the year in which the model is initialised (the earliest year that the model provides output data for). The index year could be set to correspond to the earliest year of available data, or to a policy-relevant year, e.g., the year before the policy change being modelled. The "synthetic population" in the index year is the population sample of individuals aged from the youngest age in the model (e.g. the legal drinking age in the UK of 18 years) up to a maximum age of 89 years. That sample of individuals provides the starting distributions of tobacco and alcohol consumption according to age, sex (m/f) and socio-economic conditions in terms of Index of Multiple Deprivation Quintiles (IMDQ). The number of individuals in the synthetic population in the index year can be controlled by a parameter set prior to model initialisation. The source population sample is then sampled in a bootstrap process with replacement until the specified number of individuals have been obtained. For example, a model might be initialised by sampling 200,000 individuals from a source sample of respondents to the Scottish Health Survey pooled across years 2016 to 2018.

The set of variables retained in the synthetic population are those that are needed to inform the simulation. The core variables for a model of tobacco consumption are age (in single years), sex, IMDQ, the smoking status of individuals (current smoker, former smoker, never smoked), the years since former smokers quit smoking, and amount consumed split across the two common types of tobacco product in the UK - factor-made cigarettes (FM) and handrolled tobacco (HRT).

1.5.2 Random number seeds

To minimise noise in the Monte Carlo simulation between the control and intervention arms, individuals are assigned a set of random seeds that minimise the random differences between the two arms. Everyone who enters the population simulation is assigned their own random number seed. Separate seeds are given for each process, e.g., a seed for simulating survival and a different seed for simulating transitions in alcohol consumption. The random seed for each individual is updated in each year of the simulation.

2. Data

This section describes the use of data and parameters from published literature to inform the simulated population and outcomes.

2.1 Population size data

The data on mid-year population sizes for England was supplied by the Office for National Statistics. The data cover years from 2008 to the latest available. The counts are stratified by IMD quintiles, sex, age groups by single year of age then 90+ years.

Two forms of population count data are used: (1) the observed population stratified by age, sex and IMD quintiles; (2) the forecast population stratified by age and sex. For future years, population count data by age and sex are taken from the national primary population projections. An IMD quintile split is introduced into the forecast population counts by age and sex based on the IMD quintile split in the last year of observed data.

The population count data are used to calculate model inputs of the observed population rates of death, disease, and associated hospital admissions. The population count data are also used to inform the structure of the synthetic population with which the model is initialised. Population count data for the youngest age in the model (e.g. age 18) are used to inform the number of individuals who are added to the simulation in each year when a new birth cohort is added to the model. The number of new individuals added at the youngest age in each year is the number of individuals of that age expected to be in the population multiplied by a factor calculated for the year in which the model was initialised that scales between the actual and simulated population sizes.

2.2 Health Survey for England

Population survey data is used to inform the starting distribution of alcohol consumption among individuals who enter the microsimulation, and the subsequent dynamics of alcohol consumption considering trends over age, period, and birth cohort. For England, data is used from the Health Survey for England (HSE), a series of annual surveys covering health and health-related behaviours.

2.2.1 Drinking data

The average amount drunk in a week is estimated in terms of UK standard units of alcohol (1 unit = 10ml or 8g pure ethanol). Units are estimated separately for six beverage types within the HSE: normal beer or cider, strong beer or cider, spirits, sherry, wine, and alcopops (also known as "ready to drink" or RTD). These are then grouped into four categories: beer (combining normal beer, strong beer), wine (combining wine and sherry), spirits, and alcopops. Data on the proportion of total alcohol purchasing accounted for by each beverage type in each population subgroup from the Living Costs and Food Survey (LCFS) is subsequently used to inform the splits of beer into beer and cider, and to split alcohol consumption between the on- and off-trade.

2.2.2 Smoking data

Cigarette smoking status is quantified in terms of self-reported data on whether an individual is a current, former, or never regular cigarette smoker, and if a former smoker how many years have passed since quitting. For current smokers, the number of cigarettes smoked per day is recorded. Smoking states are defined as follows:

- A never smoker is someone who has never smoked or has only tried a cigarette once or twice in their lifetime.
- A current smoker is someone who smokes cigarettes either regularly or occasionally.
- A former smoker is someone who used to smoke cigarettes either regularly or occasionally.

2.3 The Living Costs and Food Survey

The Living Costs and Food Survey (LCFS) is a repeated cross-sectional survey of roughly 12,000 households randomly sampled from across the UK. The LCFS collects transaction-level information on spending patterns and the cost of living that reflect household budgets, using a 14-day diary of all spending by household members aged 16 or over. It is conducted throughout the year, across the whole of the UK. Transaction-level data from the LCFS is accessed securely through the UK Data Service Secure Lab. LCFS data are used to produce the price distributions with which the TAX-sim model is initialised. The procedure for deriving the price distributions is as follows:

- The LCFS transaction-level data was cleaned and adjusted to 2016 prices. Each transaction was assigned a value and labelled if it fell into one of the 12 product categories of interest: beer, cider, wine, spirits, RTDs (for the on and off trade), factory made cigarettes, and handrolled tobacco.
- Each product was assigned into price per unit bins – 2p bins for tobacco and 10p bins for alcohol. For alcohol, a unit is 10ml/8g of ethanol or one UK unit of alcohol. For tobacco, a unit is one cigarette, or 0.5g of tobacco.
- Individuals are allocated to socio-demographic subgroups.

```
- Age: This is split into 4 categories: 18-24, 25-34, 35-49, 50+
- Sex: Male and Female
- Income quintile: Using the variable equivalised household income we created income
quintiles labelled 1 to 5 where 1 was the poorest households and 5 was the richest in th
e entire sample.
- Alcohol drinker category (4 groups): Abstainer, Moderate drinker (purchased 0< to 14
 units of alcohol), Hazardous (14-50 units for men, 14 -35 for women), and Harmful drin
ker (50+ for men and 35+ for women).
- Smoker category (5 groups): Non-smoker, smokes 0 to 70 a week, 70 – 140 a week, 14
0 – 280 a week, and 280+.
```

An ordered logit regression model was estimated for each of the 12 products to model the probability that a transaction occurred within a particular 10 pence price band for alcohol and 2 pence price band for tobacco. The regression model includes respondent's age-sex group, income quintile, drinker type (abstainer, moderate, hazardous, harmful) and smoker type (non-smoker, light, medium, and heavy smokers). The equation is therefore:

$$Prob(p_{it}) = f(Age_i, Sex_i, Income_i, AlcStatus_i, TobStatus_i)$$

The model predicts the probability that the price paid is within a 10p or 2p band. Subscripts i, t, denote individual i making transaction t. Each model was estimated for 50 price bands.The estimated coefficients were used to "predict" the shape of the price distributions for each subgroup defined by age, sex, income quintile, drinker status, and smoker status. This was estimated individually for the 12 products.

2.4 Mortality data

The model contains a total of 84 health conditions which are attributable to either alcohol, tobacco, or both. "Acute" alcohol-related health conditions are related to high levels of alcohol consumption on single drinking occasions. For each of the tobacco and/or alcohol related health conditions, death counts, stratified by age, sex, and Index of Multiple Deprivation (IMD)

quintile for England were obtained from the Office for National Statistics (ONS). To address year-on-year variance in small counts, we smoothed the time trends across several years and used the smoothed figure as the model input.

The mortality rate is quantified in terms of cause-specific central rates of death in one-year intervals of age and period, stratified by sex and IMD quintiles. These central death rates are converted to the probabilities of death in a one-year age interval to simulate individual deaths. They are also used to construct period lifetables from which the remaining expected years of life at each age are calculated to estimate the years of life lost to death in the STAPM simulation.

Conditions wholly or partially attributable to alcohol:

- Breast cancer
- Hypertensive diseases
- Cardiac arrhythmias
- Cirrhosis of the liver (excluding alcoholic liver disease)
- Acute pancreatitis
- Chronic pancreatitis
- Epilepsy and status epilepticus
- Transport injuries
- Fall injuries
- Exposure to mechanical forces (including machinery accidents)
- Drowning
- Fire injuries
- Other unintentional injuries
- Intentional self-harm
- Assault
- Other intentional injuries
- Accidental poisoning by exposure to noxious substances
- Alcoholic cardiomyopathy
- Alcoholic liver disease
- Acute pancreatitis (alcohol induced)
- Chronic pancreatitis (alcohol induced)
- Alcoholic gastritis
- Alcohol-induced pseudo-Cushing's syndrome
- Acute intoxication

- Mental and behavioural disorders due to use of alcohol
- Degeneration of the nervous system due to alcohol
- Alcoholic polyneuropathy
- Alcoholic myopathy
- Maternal care for suspected damage to foetus from alcohol
- Excessive blood level of alcohol
- Toxic effect of alcohol
- Alcohol poisoning
- Evidence of alcohol involvement determined by blood alcohol level

Conditions wholly or partially attributable to tobacco:

- Lung cancer
- Nasopharynx and sinonasal cancer
- Oesophageal AC cancer
- Stomach cancer
- Kidney cancer
- Lower urinary tract cancer
- Bladder cancer
- Cervical cancer
- Acute myeloid leukaemia
- Peripheral arterial disease
- Abdominal aortic aneurysm
- Venous thromboembolism
- Ulcerative colitis
- Parkinson's disease
- Alzheimers disease
- Vascular dementia
- All-cause dementia
- Depression
- Schizophrenia
- Bulimia
- Psychosis

- Multiple sclerosis
- Systematic lupus erythematosis
- Low back pain
- Psoriasis
- Age-related macular degeneration
- Crohn's disease
- Hip fracture
- Rheumatoid arthritis
- Chronic kidney disease
- End-stage renal disease
- Senile cataract
- Hearing loss
- Chronic obstructive pulmonary disease
- Asthma
- Obstructive sleep apnoea
- Idiopathic pulmonary fibrosis

Conditions wholly or partially attributable to both alcohol and tobacco:

- Oral cavity cancer
- Pharyngeal cancer
- Laryngeal cancer
- Oesophageal SCC cancer
- Oesophageal cancer
- Pancreatic cancer
- Cancer of the liver and intrahepatic bile ducts
- Colorectal cancer
- Ischaemic heart disease
- Haemorrhagic stroke
- Ischaemic stroke
- Type II diabetes
- Tuberculosis
- Pneumonia

- Influenza (clinically diagnosed)
- Influenza (microbiologically confirmed)

2.5 Hospitalisations data

For each of 84 tobacco and/or alcohol related health conditions, hospital admission rates stratified by age, sex, and Index of Multiple Deprivation (IMD) quintile are calculated from individual patient records taken from the admitted patient care data from Hospital Episode Statistics. Rates of hospital admission can be calculated using different methods (see the discussion paper that we developed to understand the range of methods).

The version of the STAPM model used in this study uses a version of the "narrow" method to estimate rates of hospital admissions. The narrow rate is a more specific estimate of the rates of hospital admission for each condition per 100,000 people. The narrow rate looks only for tobacco and/or alcohol related diagnosis codes in the primary diagnostic position of an episode of care. Subsequent diagnostic positions are also scanned for external cause codes, e.g., assault, which do not feature in the primary diagnostic position but are important elements of the list of tobacco and/or alcohol related conditions considered in the model.

Two outcomes are produced from the hospital record data:

1. Morbidity rates per 100,000 (derived using NRS population estimates) for each health condition and age-sex-socioeconomic status subgroup. Year-on-year variance in small counts is addressed by smoothing the time trends in the calculated rates.
2. Unit costs of hospitalisations by condition, age group, sex and IMDQ. These costs are derived from the hospital episode-level HRG reference costs associated with the length of stay in hospital and the procedures applied. Currently our unit costs are calculated based on the 2016/17 data. Unit costs are inflated as necessary using the Hospital and Community Health Services (HCHS) pay and price inflation index.

3. Microsimulation of tobacco consumption dynamics

3.1 Smoking participation

The STPM model is fundamentally based upon what happens to an individual's smoking status over time, and the resulting risk of disease and mortality for the individual concerned. Smoking status of an individual i, who is a member of population subgroup j, and has age a, in year y is categorised as either a never smoker, a current smoker, or a former smoker. The figure below shows the transitions modelled in the simulation.

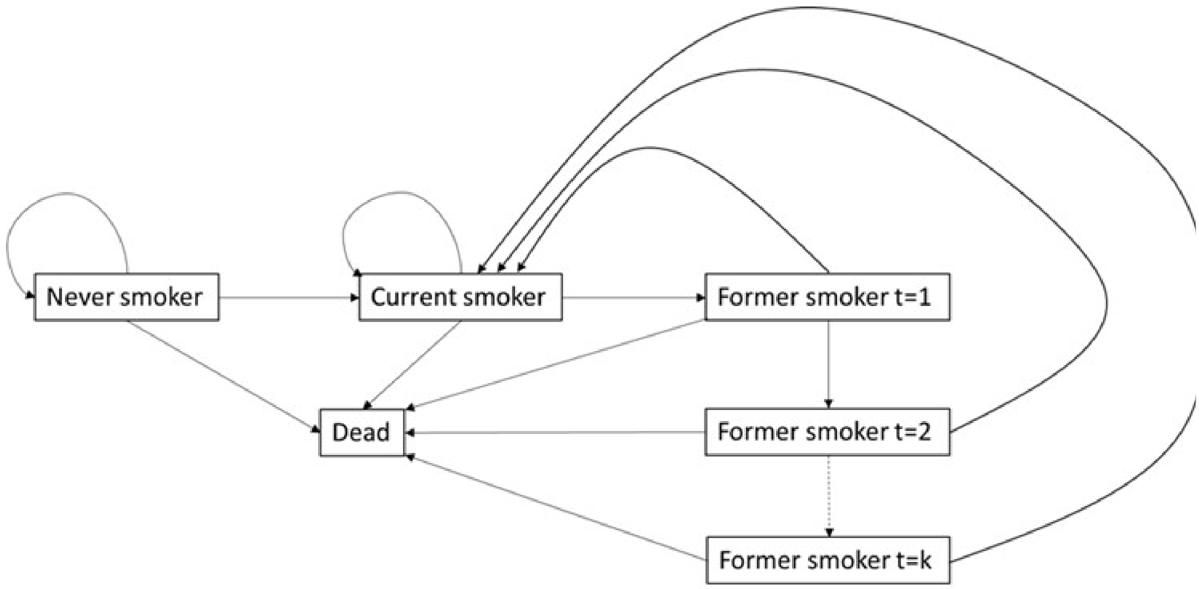

Figure 3.1: Transitions in smoking status in the model

Transitions in smoking status are modelled and updated annually. An individual who is a never smoker at year y, can then either transition at year $y + 1$ to being a current smoker because they have initiated regular smoking tobacco, or they can remain a never smoker at $y + 1$. An individual who is a current smoker at year y, can then either transition at year $y + 1$ to being a former smoker because they have quit tobacco, or they can remain a current smoker at $y + 1$. An individual who is a former smoker at year y, can then either transition at year $y + 1$ to being a current smoker because they have relapsed and begun to smoke tobacco again, or they can remain a former smoker at $y + 1$. For a former smoker, the simulation also tracks how many years it is since they quit smoking, denoted by x_i, so that as each year ticks forward and y moves to $y + 1$, then the x_i moves to $x_i + 1$. In the current version of the model, the probabilities of relapse to current smoking in former smokers are highest in the first year after quitting, and then decline up to 10 years post-quitting, after which they remain constant.

From a birth cohort perspective, the model describes how the number of individuals alive at the start of each year, denoted A, changes as the years move forward and people age and as they transition between the possible smoking states. The proportions of people by smoking status in birth cohort b at each age a and year y, for each subgroup j, are denoted as follows:

- $\theta_{b,n}(a, y, j)$, is the proportion of people alive in the birth cohort who are never smokers
- $\theta_{b,c}(a, y, j)$, is the proportion of people alive in the birth cohort who are current smokers
- $\theta_{b,f}(a, y, j)$ is the proportion of people alive in the birth cohort who are former smokers.

For former smokers, the model tracks the number of years (x) that they have spent as a former smoker; the proportion of former smokers with each number of years as a former smoker is f_x.

If $A_b(a,y,j)$ is the number of individuals alive in the birth cohort at age a, then the corresponding number of people alive in the birth cohort at age a and who are current smokers is

$$A_{b,c}(a,y,j) = A_b(a,y,j)\theta_{b,c}(a,y,j) \qquad (3.1)$$

The number of current smokers at age $a+1$ years in year $y+1$ depends on the addition of new smokers from the never or former smoking states (through the probabilities of initiation and relapse respectively), and on the removal of smokers through the probabilities of quitting or death.

The model describes four main probabilities for people - initiating smoking, quitting smoking, relapsing back to smoking, and survival to the next year.

- $P_n(initiate|a,y,j)$ is the age-specific probability of smoking initiation, i.e. that an individual who is observed as a never smoker at age a years will be observed as a current smoker at age $a+1$ years.
  $P_c(quit|a,y,j)$ is the age-specific probability of quitting smoking, i.e. that a current smoker at age a years is observed as a former smoker at age $a+1$ years.
- $P_f(relapse|a,x,y,j)$ is the probability of relapse from former smoking to current smoking for an individual aged a years and with a certain number of years since quitting.
- $P(survive|a,y,j)$ is the probability of survival from age a to $a+1$ years, and is conditional on age, year, population subgroup and smoking status.
- $P_n(survive|a,y,j)$ is the probability of survival of never smokers.
- $P_c(survive|a,y,j)$ is the probability of survival of current smokers.
- $P_f(survive|a,y,j)$ is the probability of survival of former smokers.

The number of current smokers at age $a+1$ years can then be written as the sum of three terms, i.e. the current smokers who survive and don't quit, plus the former smokers who survive but relapse back to smoking after several years, and finally plus the never smokers (up to age a) who initiate:

3.1.1 Period dynamics of smoking

Based on formula for the number of current smokers and related formulae for the numbers of never and former smokers, the model describes how the population-level distribution of individuals among smoking states observed in time period (y, e.g. $y =$ the year 2001) changes over calendar time. The mathematical formulation below draws inspiration from the mathematical description of the dynamics of quantitative traits in age-structured populations by Coulson & Tuljapurkar[4].

The number of current smokers in year y is

$$A\_c(y,j) = \sum_{a=a^{min}}^{a\_max} A\,(a,y,j)\theta\_c(a,y,j) \qquad (3.2)$$

The number of current smokers in year $y + 1$ depends on the change to the number of smokers in each birth cohort (where $b = y - a$), and on the turnover of birth cohorts, i.e. the loss of old birth cohorts as they age and die out, and the entry of new birth cohorts into the population. The dynamics of population size are tracked by using data on the time trend in the number of people at the youngest age $A(a_{min}, y, j)$. The number of current smokers at $y + 1$ can then be written as

where the first line are the current smokers who do not quit, the second line are the former smokers who relapse, the third line are the never smokers who initiate, the fourth line are the members of the new cohort who arrive at the youngest age of a_{min} years, and the fifth line are the individuals who reach the oldest age in the model of a_{max} years and who all die in the next year.

3.1.1.1 Approach to extrapolating future smoking state transition probabilities

A central idea of the STPM model is that it can extrapolate the recent trends in smoking prevalence into the future based on separate extrapolations of the probabilities of smoking initiation, quitting and relapse. As a result of having this functionality, STPM has the ability to compare among alternative future scenarios, e.g. what would happen if the probabilities of quitting continued to increase along their recent trajectory vs. staying constant, or what if the probabilities of initiating smoking began to increase vs. decrease. Below we describe the method used to extrapolate our estimates of smoking state transition probabilities into the future.

The smoking state transition probabilities of smoking initiation, relapse and quitting are extrapolated along separate trajectories of calendar time for each sex and IMD quintile, i.e. we produce ten separate extrapolations for each of smoking initiation, quitting and relapse.

We use a forecasting method based on the Lee-Carter approach[5]. Our method first smooths and logit transforms the age-period surface of the estimated smoking state transition probabilities, and then based on a singular value decomposition estimates the overall rate of change over time and the age-emphasis of this change, which are then projected into the future.

As with any forecast, the predicted future trends are sensitive to the time period of past trends that are used to inform the forecast, e.g. the predictions might vary depending on whether the observed trends over the past 5 vs. 10 years are used. The choice of time period might be constrained by the available data, or it might be affected by an understanding of relevant changes to society, e.g. quitting might be informed by trends since 2013, when e-cigarette use began to increase.

3.1.2 Dynamic changes to consumption of tobacco for smokers

This section describes the method to track the life-course dynamics of the average amount of cigarettes that they consume per day by individuals who remain as current tobacco smokers between years of the microsimulation. When an individual enters the simulation as a smoker, either in the starting population sample or if they initiate smoking or relapse to smoking, they are assigned an amount smoked based on the distribution of amount smoked in the current year, according to their age, sex and IMDQ.

Individuals who remain as current smokers between successive years of the simulation have the amount that they smoke updated to represent the expected change in amount smoked

over age. The assumption applied in the structure of the STAPM model is that each current smoker remains at approximately the same percentile of the distribution of amount smoked as they age, i.e., the model simulates change to the population distribution of amount smoked with age, but the model does not simulate random movements of individuals between points in the distribution as they age.

In the current version of STAPM, change to the shape of the distribution of amount smoked with age is estimated from a HSE dataset for ages 13-89 and years 2013-2018, stratified by sex and IMDQ. The shape of the distributions of the average number of cigarettes per day smoked by current smokers is described by empirical cumulative distribution functions (ecdf) corresponding to each calendar year, single year of age, sex and IMDQ. A sample of 200,000 individuals is generated from the HSE data sample by bootstrap re-sampling. Estimates of the distribution of amount smoked are then obtained as 3x7 year rolling average distributions by year and age. A 'trend limit' is imposed such that there is no change in the reference distributions by calendar year. This limit means that the only variation in tobacco consumption that is modelled is that between ages for the index year (2017).

The update of the average number of cigarettes smoked per day by individuals who remain as current smokers as they age cycles through each single year of age for each sex and IMDQ subgroup, as follows:

Define the donor values and percentiles – Define a 'mesh' of possible values of consumption that an individual could transition to at age a+1. For the SYNTAX modelling, the mesh for the average number of cigarettes smoked per day was a sequence of length 1,200 that ran from 1/365 to 60, which gave a mesh width of approximately 0.05 cigarettes per day. Using the ecdf of consumption corresponding to age a+1, each value in the mesh is assigned a percentile in the distribution of consumption. This produces a 'donor' set of percentiles corresponding to the possible new values of consumption.

Define the reference values and percentiles - Look at the average number of cigarettes consumed per day by current smokers in the simulated population at age a. Using the ecdf of consumption corresponding to age a, current smokers are assigned a percentile in the distribution of consumption. This produces a 'reference' set of percentiles for the current smokers at age a.

Match the donor and reference percentiles and update individual consumption - For current smokers aged a, determine the nearest neighbour match between their reference percentile and the donor percentiles. Assign individuals the new value of the average number of cigarettes smoked per day at age $a + 1$ that corresponds to the selected donor percentile.

3.1.3 Estimating change to the preference between factory-made and handrolled cigarettes

Change to a 2-value preference vector that gives the proportional split of the average number of cigarettes smoked per day between factory-made (FM) cigarettes and roll-your-own (RYO) cigarettes is simulated as current smokers age. The new preferences for age $a + 1$ are sampled from the distribution of preferences of the smokers in the current year of the microsimulation within each sex and IMDQ subgroup. This means that the subgroup distributions of product preferences are preserved between years of the microsimulation but that each current smoker moves at random within the distribution of preferences for their sex and IMDQ subgroup as they age.

4. Modelling the relationship between consumption and harms

An epidemiological approach is used to model the relationship of changes in tobacco and alcohol consumption to changes in mortality and morbidity outcomes. The changes in tobacco and alcohol consumption that are defined within the model to affect mortality and morbidity are:

- Changes in smoking state, with lagged effects of quitting smoking.
- Changes in average weekly amount drunk, with lagged effects of changes in consumption.

4.1 Relative risks of disease

STAPM considers the effects of tobacco and alcohol consumption on the risks of developing the ICD-10 defined categories of disease (Section 2.4). In each year of the simulation, individuals in the simulated population are assigned a relative risk of disease based on their smoking and drinking behaviour and history, with risks differentiated by age and sex where this information in available.

The alcohol modelling considers 45 categories of adult diseases related to alcohol consumption and the corresponding dose-response effects of current levels of alcohol consumption on the relative risks of disease. The model links average weekly alcohol consumption to the risk of chronic diseases, and the amount of alcohol consumed on single drinking occasions to health harms associated with intoxication. Individuals' relative risk for each disease is calculated for four categories of disease:

- Chronic diseases partially attributable to long-term average levels of alcohol consumption – see Angus et al.[6] for the list of diseases, sources of risk functions and their corresponding curves. For some conditions there are separate risk functions for mortality and morbidity. For conditions that show a J-shaped risk function this indicates protective effects of alcohol for levels of alcohol consumption where the relative risk is less than 1.
- Chronic diseases wholly attributable to long-term average levels of alcohol consumption (Section 4.1.1).
- Diseases partially attributable to acute alcohol consumption (Section 4.1.2).
- Diseases wholly attributable to acute alcohol consumption (Section 4.1.3). See Section 4.1.4 for how the lagged effects of changes in consumption on the risk of chronic diseases are computed.

The smoking modelling considers 52 categories of diseases related to smoking in adult smokers and the corresponding relative risks of these diseases in current vs. never smokers, and in former smokers according to the time since they quit[7]. See Section 4.1.5 for how the lagged effects of quitting smoking on disease risks are computed.

There are 15 diseases in our combined list of 84 diseases that are related to both alcohol and tobacco:

- Cancers of the oral cavity, pharynx, larynx, oesophagus, pancreas, liver, colon & rectum.
- Cardiovascular diseases - ischaemic heart disease, haemorrhagic and ischaemic stroke.
- Type II diabetes.
- Tuberculosis, pneumonia, and influenza.

For oral cavity, pharynx, larynx and oesophageal cancers there is information from meta-analyses of interactions between tobacco and alcohol disease risk. This evidence shows that the risk of disease in someone who consumes both tobacco and alcohol is higher than would be expected from combining the independent risks from tobacco and alcohol. This additional risk due to tobacco-alcohol interaction is expressed as a "synergy factor"[8,9]. The effects of including the synergy factors are not included in the base-case results of the STAPM modelling – in the base-case the relative risks for drinking and smoking are combined as follows:

$$RR_{combined} = 1 + \left((RR_{alcohol} - 1) + (RR_{tobacco} - 1)\right)$$

In sensitivity analyses, synergistic effects are included as follows:

$$RR_{combined} = 1 + \left(\left((RR_{alcohol} - 1) + (RR_{tobacco} - 1)\right) * SynergyFactor\right)$$

4.1.1 Chronic diseases wholly attributable to long-term average levels of alcohol consumption

The diseases in this category are: Alcoholic cardiomyopathy, Alcoholic gastritis, Alcoholic liver disease, Acute pancreatitis alcohol induced, Chronic pancreatitis alcohol induced, Alcohol induced pseudo-Cushing's syndrome, Alcoholic myopathy, Alcoholic polyneuropathy, Maternal care for suspected damage to foetus from alcohol, Degeneration, Mental and behavioural disorders due to use of alcohol[6].

STAPM uses the SAPM method to determine the shape of the risk function (the method is described on p28 in the Purshouse et al.[10] modelling report for NICE). Briefly, for wholly attributable chronic conditions, risk is defined as the difference between mean daily consumption and a lower threshold below which risk is assumed to be equivalent to that of abstainers. The thresholds used are 2 units/day for both females and males (equivalent to 14 units/week). Below these thresholds risk is assumed to be 0.

4.1.2 Diseases partially attributable to acute alcohol consumption

Diseases in this category are injuries, e.g., traffic accidents, assault and falls, that are linked to the amount consumed on single drinking occasions and the subsequent amount of time that individuals remain intoxicated with a percentage blood alcohol content (%BAC) greater than zero. The STAPM method to calculate the relative risk of injuries for each drinker builds on the method used in SAPM4 - detailed description of the method can be found elsewhere[11,12]

Calculate the patterns of single occasion drinking for individuals in the simulation - In each one-year time step of the STAPM simulation, individuals' average weekly alcohol consumption is converted to estimates of:

- The frequency of drinking occasions (defined as *n*, or number of drinking occasions per week)

- The distribution of the amount of alcohol consumed on each occasion – this is a vector of probabilities that each integer number of grams of ethanol per day from 1-600g/day is consumed on a single drinking occasion. For each individual, alcohol consumption on a given drinking occasion is assumed to follow a normal distribution with mean of μ and standard deviation of σ. It is computed from the:
    - mean level of alcohol consumption for a given drinking occasion (defined as μ, or units of alcohol)
    - the variability of alcohol consumption for a given drinking occasion (defined as σ, or standard deviation of units of alcohol consumed in drinking occasions).

The parameters used in the calculation of patterns of single occasion drinking come from Hill-McManus et al.[12], who analysed drinking occasions using data from detailed diaries in the National Diet and Nutrition Survey 2000/2001. The parameters allow prediction of an individual's characteristics of single occasion drinking from their average weekly alcohol consumption and a range of other characteristics that include age, income, employment, ethnicity, age left education, and social class. It was necessary to get the parameters into a form in which they fit with the age, sex and IMDQ stratification of the STAPM model. To do so, an additional computation was conducted to prepare the parameter inputs to the STAPM model - the parameters from Hill-McManus were assigned to individuals in the HSE 2011-2017 based on the full range of individual characteristics included in the statistical models. Weighted averages of these parameters were then calculated to give STAPM model inputs for each age category, sex and IMDQ.

Estimate annualised relative risk of alcohol-attributable injuries for individuals in the simulation - The duration of intoxication corresponding to the amount of alcohol drunk on an occasion is defined in terms of the time, in hours, after a drinking occasion that it would take for an individual's %BAC to drop to zero. The calculation of this time considers individuals' sex, height, and weight[13,14]. The rate at which the liver clears alcohol from the body is assumed to be 0.017 %BAC per hour. The probability that each level of alcohol is consumed on a drinking occasion is multiplied by the expected duration of intoxication for each amount of alcohol drunk on an occasion. The result of that calculation is then multiplied by the expected number of drinking occasions per week and by 52 weeks in a year. The annualised relative risk is calculated by summing the relative risk of injury across the year - the time during the year that is spent intoxicated is associated with the relative risks for the amount drunk on the occasion from Cherpitel et al.[15], and the remaining time in the year that is not spent intoxicated has a relative risk of 1.

4.1.3 Diseases wholly attributable to acute alcohol consumption

Diseases in this category are those that can only be caused by acute alcohol consumption, e.g., alcohol poisoning.

The STAPM method to calculate how the risk of these acute harms varies with the level of consumption on a single drinking occasion builds on the SAPM method – detailed description of the method can be found on page 29 in the Purshouse et al. modelling report for NICE[10]. Due to the harms being wholly attributable to alcohol, no cases are expected in people who consume below a certain threshold of alcohol on a single occasion, i.e., there are no cases in the non-exposed reference group. The starting point for the computation is the probability distribution over the number of units of alcohol that could be consumed on a single drinking occasion (Section 4.1.2). Values in the probability distribution corresponding to numbers of

units below the binge drinking thresholds (3 units a day for women, 4 units a day for men) are set to zero. The probability distribution is then used to compute the total number of units above the thresholds expected to be drunk in a year. Risk is assumed to be proportional to that value.

4.1.4 Lag times from changes in alcohol consumption to changes in chronic disease risk

When modelling the link between alcohol consumption and the risk of chronic disease over many years, an important input is the assumption surrounding the 'time lag' – the time needed to achieve the full effect of a change in consumption on a change in disease risk. SAPM4 uses estimates of the temporal relationship between changes in consumption and the proportional reduction in the relative risk of disease, assuming that the full impact of a change in consumption on risk occurs by 20 years after the change in consumption (Holmes et al.[16]). The SAPM4 method needed to be adapted for STAPM to suit the STAPM model structure of simulating individual life-course trajectories of alcohol consumption. This meant that adding memory to track individual histories of relative risk for each disease over their life-courses.

STAPM stores individual risk histories for up to 20 years. In each year of the simulation, the stored risk histories are filtered to retain only the individuals currently present in the simulation. The relative risk of each disease assigned to an individual in the current year of the simulation is adjusted to take account of the stored risk history. For each annual stored risk value, the time difference to the current year of the simulation is calculated and the corresponding proportional reduction in the relative risk of disease is merged into the data. The adjusted relative risk of disease for the current year of the simulation is computed as a weighted average of the relative risks for past years for which the individual was tracked, where the weights correspond to the proportional reduction in relative risk. This means that the relative risk for the current year always has the lowest weight, which reflects the gradual emergence of the effects of changes in consumption on the risk of disease.

4.1.5 Declines in risk over time after quitting smoking

Former smokers are initially given the relative risk associated with current smokers, which we then scale according to a disease-specific function that describes how risk declines after quitting smoking. After 40 years from quitting, we assume that risk has reached the level of a never smoker. To estimate the risk of disease for former smokers, STAPM uses the findings of Kontis et al.[17], who re-analysed the change in risk after smoking in the ACS-CPS II study from Oza et al.[18], producing three functions to describe the decline in risk after quitting for each of cancers, CVD and COPD (Figure 4.1). Kontis et al. state that "Randomised trials also indicate that the benefits of behaviour change and pharmacological treatment on diabetes risk occur within a few years, more similar to the CVDs than cancers.[19] Therefore, we used the CVD curve for diabetes." In-line with Kontis, we apply the rate of decline in risk of CVD after quitting smoking to type 2 diabetes. For other diseases, we assume that the relative risk reverts to 1 immediately after quitting i.e., an immediate rather than a gradual decline in risk.

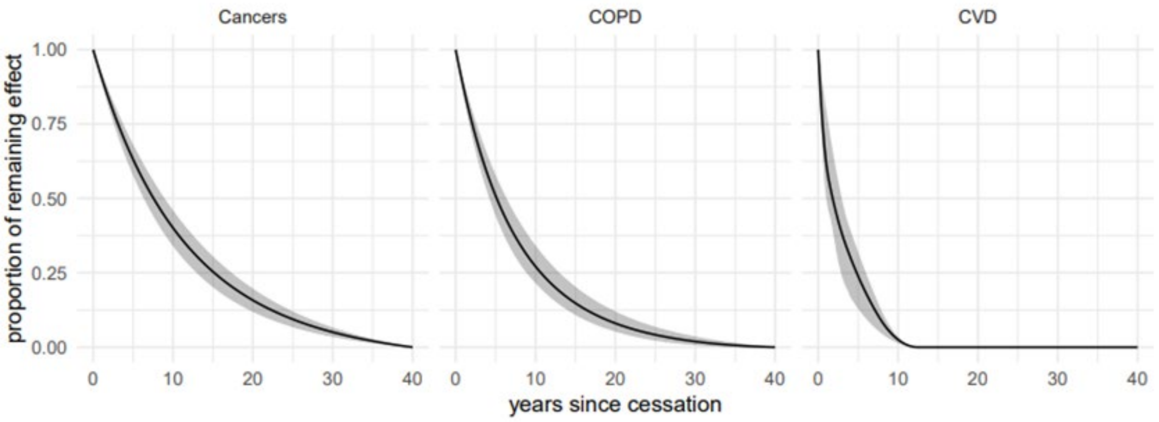

Figure 4.1: The proportion of excess relative risk remaining at each time-point since cessation

4.2 Calculating population attributable fractions to estimate the total avoidable burden of mortality and morbidity due to tobacco and alcohol in a single year

This section describes the method to estimate Population Attributable Fractions (PAFs) - the proportions of cases of tobacco and/or alcohol related diseases that could be prevented if exposure to tobacco and alcohol in the population were removed[2021].

PAFs are estimated to show the potential impact of removing exposure to tobacco only, alcohol only and both tobacco and alcohol. The joint tobacco and alcohol PAFs tend to be smaller than the sum of the tobacco only PAF and the alcohol only PAF – because some cases of disease would require the removal of exposure to both tobacco and alcohol to be prevented, i.e., single substance PAFs over-estimate by not considering the remaining influence of other exposures. The estimated effects of removing only tobacco or only alcohol are therefore adjusted downwards so that they sum to the estimated effect of removing both tobacco and alcohol.

Formula for the PAF – the formula used in this project has two inputs: (1) data on the distribution of tobacco and alcohol consumption among individuals in a cross-sectional sample; (2) estimates of how the relative risk of disease varies with tobacco and alcohol consumption (Section 4.1). It is defined for each disease as:

$$PAF = \frac{\sum_i P_i\,(RR_i - 1)}{1 + \sum_i P_i\,(RR_i - 1)} \qquad (4.1)$$

where *i* is an index of exposure to tobacco and/or alcohol, and *RR* is the relative risk of disease. PAFs might be estimated using Equation 4.1 separately for calendar years, ages or various definitions of population strata.

For some diseases, the relative risks indicate that some levels of exposure to tobacco or alcohol consumption protect against the disease, i.e., that some smokers or drinkers are less likely to get the disease. Protective effects are defined by RR < 1, which when Equation 4.1 is applied results in negative PAFs.

4.3 Linking behaviour change to change in mortality and morbidity between years in the microsimulation

This section describes how in STAPM changes in tobacco and alcohol consumption are linked to changes in mortality and morbidity rates each time the simulation "clock" ticks forwards one year. Annual updates to mortality and morbidity occur in both the control and intervention arms of the model, which means that when a tax change is applied in the intervention arm, its effects on tobacco and alcohol consumption cause mortality and morbidity divergence between model arms. The rates of mortality and morbidity that are updated are disease-specific and stratified by population subgroups defined by combinations of single years of age, sex and IMDQ.

The method applied in STAPM to link changes to consumption to changes to the rates of mortality and morbidity builds on the method applied previously in the SAPM model (see Section 2.3 in Brennan et al.[22]), which built on previous methods e.g., Gunning-Schepers[23]. All methods use the same basic method, which is based on the notion of the population-attributable fraction (Section 4.2). The rates of mortality and morbidity are updated in each year of the model simulation according to a proportional factor of change in the average relative risk of disease within each population subgroup. The proportional factor of change is the ratio of the average relative risks in the subgroup to be updated in the current year of the simulation relative to the previous year of the simulation. This proportional factor of change is known as the 'potential impact fraction' (PIF) or 'trend impact fraction'.[23]

The PIF is calculated for each disease for mortality or morbidity using the formula:

$$PIF(y,j) = 1 - \frac{RR_{average}(y,j)}{RR_{average}(y-1,j)} \qquad (4.2)$$

where y indicates the current year of the simulation and j indicates each age, sex, and IMDQ subgroup.

The rates of mortality and morbidity from each tobacco and/or alcohol-related disease are updated in each year of the model simulation according to:

$$m(y,j) = m(y-1,j)[1 + PIF(y,j)] \qquad (4.3)$$

where $m(\quad)$ is a generic notation for either the rates of mortality or morbidity.

4.4 Mortality analysis

The starting point for the analysis of mortality outcomes is disease-specific mortality, defined as central rates of death in one year intervals of age and calendar year, stratified by sex and IMDQ (Section 2.4).

Calculate individual probabilities of death – In each year of the simulation, a probability of death for each individual is calculated. This probability of death incorporates mortality from tobacco and/or alcohol related diseases and from all other diseases not related to tobacco or alcohol consumption. The calculation of individual probabilities of death is based on a method used in the SAPM model, briefly as follows:

- Assigning individuals a relative risk for each tobacco and/or alcohol related disease based on their consumption status (Section 4.1).

- Standardise the distribution of individual relative risks of disease so that they sum to 1 for each combination of age, sex and IMDQ subgroup.
- Multiply the disease-specific mortality rate for each age, sex and IMDQ subgroup by the standardised individual relative risks of disease and divide this by the subgroup average of the standardised individual relative risks.
- The result is individualised estimates of disease-specific mortality rates, which average to equal the subgroup mortality rate.
- The calculation assumes that the only cause of mortality variation among individuals within a year, age, sex and IMDQ subgroup is their tobacco and/or alcohol consumption.
- Compute the average cause-specific mortality rate according to levels of tobacco and alcohol consumption, age, sex and IMDQ – for use in reporting of model outputs.
- Convert the individualised estimates of cause-specific mortality to cause-specific probabilities of death for each individual during the year, assuming that mortality is constant during each one-year age interval.
- Sum the probabilities of death across causes – for subsequent use in simulating which individuals die in each year of the simulation.

Simulate individual deaths - The probability of death calculated for each individual is used to Monte Carlo simulate who dies by drawing from a binomial distribution, and the individuals who die are removed from the simulated population. The number of individuals who die in each year of the simulation for each age, sex and IMDQ subgroup is recorded. Those individuals still alive after the simulation of deaths then form the population for the next year of the simulation and the sequence repeats.

Calculate effects of the intervention on deaths and years of life lost to death – After the model simulation has run, 'post-processing' is conducted to compute the effects of the intervention on lifetable outcomes for:

- Number of years of life lived ("life-years") - shown by effects on the simulated population size, since the simulation moves forward in one year time intervals.
- Number of deaths in each year - estimated either from the number of deaths recorded in the model simulation, or from the estimated mortality rate and population size (the latter will be subject to less stochastic variation).
- Number of years of life lost due to deaths from tobacco-related causes - estimated by multiplying the number of deaths recorded at each age, in each sex and IMD quintile stratum by the expected remaining years of life for someone of that age in that stratum. The remaining expected years of life are therefore influenced by the modelled age- and stratum-specific mortality rates, i.e., both number of deaths and expected remaining years of life can respond to a policy change.

The effect of the intervention is then calculated as the annual or cumulative annual difference between these outcomes for the control and intervention arms.

4.5 Morbidity analysis

After the model simulation has run, 'post-processing' is conducted to compute the effects of the intervention on outcomes for numbers of hospital admissions, and NHS costs of hospital admissions.

- Number of hospital admissions
- NHS costs of hospital admissions

The methods to do so build on the approach taken by previous SAPM modelling with adaptations to ensure that policy effects on the above outcomes were comparable for tobacco and alcohol. These adaptations mean that the rates, multipliers, and unit costs used in the STAPM modelling differ slightly from those in the SAPM modelling. Where method changes were made, a conservative approach was taken such that STAPM tends to generate lower cost estimates than SAPM.

Partitioning individuals among morbidity states - The morbidity model works by partitioning the alive population in each year of the simulation between all 84 alcohol-related health states (and an 85th state representing overall population health, not attributable to tobacco or alcohol). Disease-specific morbidity is defined as "person-specific single morbidity" (PSSM) rates in one year intervals of age and calendar year, stratified by sex and IMDQ (see the report). The PSSM rates give the proportion of people in each of 84 ICD-10 defined health states. Using the methods described for mortality, individualised estimates of the PSSM rates are computed and then averaged across tobacco and alcohol consumption categories, age, sex and IMDQ subgroups for use in reporting of model outputs.

NHS costs of hospital admissions - Each hospital admission associated with a health condition is then assigned an average cost for the admission, where the costs of admissions are calculated considering only episodes of care that share a primary diagnosis with the diagnosis assigned to the admission as a whole (again, this is in keeping with our conservative approach to costing). New methods were added for STAPM that improve our estimation of the change in the NHS costs of hospital admissions by linking the Healthcare Resource Group fields to standard NHS intervention costs. Costs are inflated to 2022 prices using the Hospital and Community Health Services (HCHS) index. Costs are stratified by condition, sex and IMDQ.

5. Modelling the relationship between price and consumption

TAX-sim models the impact of pricing policies on prices and subsequent effects on consumption. The effects on consumption impact on health outcomes through the mechanisms previously described. In this section we give an overview of the policy to price component of the model and detail how estimated changes in price impact on consumption in the model. Subsequent sections describe in detail how different types of policy affect prices.

The figure below presents a schematic of the policy to price component of the TAX-sim model.

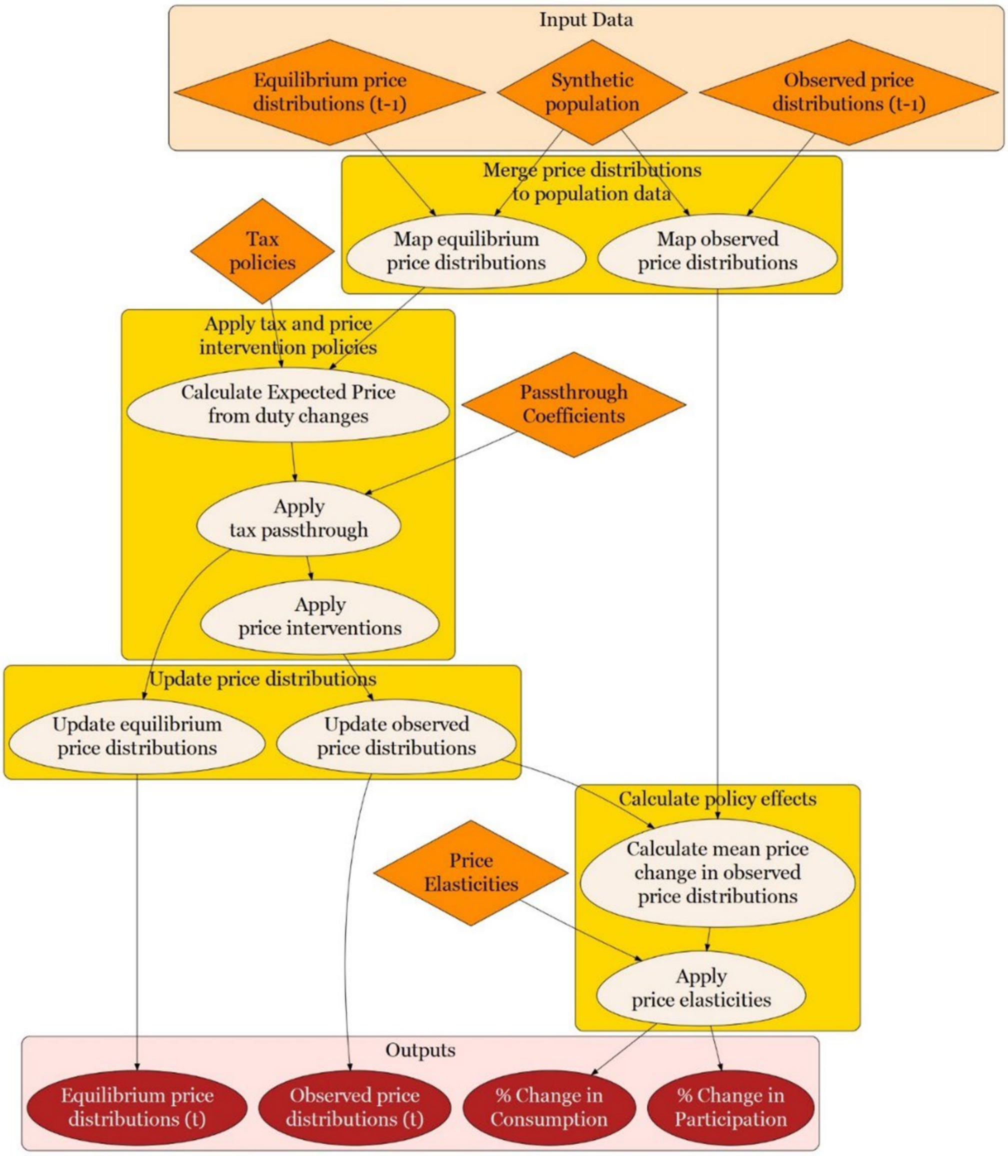

Schematic of the TAX-sim workflow for calculating price and consumption effects

5.1 Overview of policy to price modelling

In a year of the simulation, the workflow has the following steps:

1. *Match price distributions*. The synthetic population is merged to subgroup-level distributions of prices as of the end of the previous year. There are two types of price distribution - *equilibrium price distributions* reflect the distribution of prices given the current tax regime. *Observed price distributions* are price distributions which incorporate the effects of price control policies such as minimum unit pricing or wholesale price caps. In the absence of such policies the equilibrium and observed price distributions will be identical.

2. *Update the equilibrium price distribution*. Updated rates of specific duty, ad-valorem duty, and/or value-added tax (VAT) are applied to the equilibrium price distribution to calculate the expected price. The expected price is the price that keeps retailer net revenue constant following the tax policy change.

3. *Apply tax passthrough*. Tax passthrough in the model is based on empirical estimates of industry behaviour. To estimate passthrough coefficients, quantile regressions are estimated at the mid-point of each decile of the observed price distribution, with observed price as the outcome variable and expected price as the independent variable. The tax passthrough coefficients are used to calculate the new observed price. The coefficients used represent the most up-to-date empirical estimates for off-trade alcohol[24], on-trade alcohol[25], and tobacco[26].

5.2 Price distributions

To model the effects of a tax change, it is necessary to assign individuals in the simulation a distribution of prices paid for tobacco and alcohol products. To do this, individuals are probabilistically assigned to a distribution of prices paid based on their age, sex, income, tobacco consumption and alcohol consumption. This is done so we can understand the mean price that each individual pays for a product, the tax breakdown of these prices and how the mean prices paid change due to a policy intervention.

The price distributions in the model are subgroup level and split by age group, sex, income quintile, drinker category, and smoker category. Price distributions are characterised as population subgroup j's consumption of product k by dividing the distribution into 50 transaction price bands and recording the proportion of total spending on product k by subgroup j occurring at transaction price band l.

All transactions in a price band are assumed to occur at the price representing the mid-point of the band i.e. purchases between 50 and 59p per unit of alcohol (where a unit of alcohol is defined as 10ml/8g of pure ethanol) are set at 55p. Alcohol price distributions are defined over the range 0 - 500p per unit in 10p intervals, and tobacco price distributions are defined over the range 0 - 100p per stick in 2p intervals. A stick is defined as one factory-made cigarette or 0.5g of hand-rolling tobacco.

The average price an individual pays for a particular product is summarised as the mean of the price distribution they face, based on their subgroup, calculated as the mean of each price point weighted by the share, δ_{jkl}, of total consumption of product k by subgroup j occurring at transaction price l:

The price distributions are merged into the simulated dataset in two steps:

(1) Since income quintile is not in the starting population, nor do we explicitly model the transition of income over time, the first step is to probabilistically assign individuals to one of 5 income quintiles based on their current age-band (18-24, 25- 34, 35-54, 55+), sex, IMDQ, tobacco consumption (5 levels) and alcohol consumption category (4 levels). The method to generate the probabilistic map between IMDQ and income quintiles uses 6 years' worth of data for HSE data from 2013 to 2018. This produces a look-up table that gives the probability an individual is in each of the 5 equivalised income quintile categories. In each year as the simulation progresses, the individual is Monte Carlo resampled as to their income quintile based on their updated characteristics.

(2) Merge the price distributions with the simulated individual dataset based on their simulated age, sex, income quintile and tobacco and alcohol consumption. Merging the price distribution with the simulated individual dataset in each year of the simulation ensures that

the price distributions assigned to individuals in the microsimulation can vary over individual life courses, i.e., if an individual ages or changes their level of tobacco or alcohol consumption during the simulation.

Price distributions are expressed in real-terms values, adjusted for inflation by the Retail Price Index (RPI). All specific duty rates per unit and price policy parameters are also expressed in real-terms, and so it is assumed that it is **real terms changes in policy parameters** that have an impact on the price and so on consumption.

5.3 Price elasticities of demand

The price elasticities of demand model the effect on whether an individual consumes a product at all (participation), and for those that are consumers of a product, how much of it they consume (conditional consumption). Estimates of consumer responses to price changes take the form of 'price elasticities of demand', or the proportional change in consumption for a unit proportional change in price. The 'own-price' elasticity tells us the response of demand for a product to a change in its own price. The 'cross-price' elasticity tells us the response due to a change in another product's price.

There are two types of elasticity - participation and conditional consumption. Participation elasticities measure the percentage change in participation (the probability of an individual consuming, or the proportion of a subgroup who are consumers) for a percentage change in price. Conditional consumption elasticities measure the percentage change in the amount consumed by consumers for a percentage change in price. Elasticities are entered into the model as a set of square matrices (one for participation and one for conditional consumption) with 12 rows and columns to account for each of the 10 alcohol and 2 tobacco product own-price and cross-price elasticities which apply to all individuals in the population.

There is a substantial literature on the estimation of price elasticities for alcohol[27] and for tobacco[28] separately, but little evidence on joint estimation of tobacco and alcohol price elasticities. For the UK, there are no studies in which a single econometric modelling approach is used to estimate joint price elasticities for participation in, and consumption of, both tobacco and alcohol products simultaneously.

The element e_{k_x,k_y} in Equation 5.1 represents the elasticity of demand for product y with respect to the price of product x. For $x = y$ on the lead diagonal of the elasticity matrix, e is the own-price elasticity - the responsiveness of consumption of a product to changes in its own price. Off-diagonal elements are cross-price elasticities, e.g. $e_{1,2}$ is the elasticity for a change in consumption of product 2, given a 1% increase in the price of product 1.

$$\epsilon = \begin{pmatrix} e_{1,1} & e_{1,2} & \cdots & e_{1,12} \\ e_{2,1} & e_{2,2} & \cdots & e_{2,12} \\ \vdots & & & \vdots \\ e_{12,1} & e_{12,2} & \cdots & e_{12,12} \end{pmatrix} \qquad (5.1)$$

As proportional changes to product price in the model are stratified by population subgroup, proportionate changes in participation and conditional consumption are calculated for each population subgroup. These proportionate changes are then applied to individual level consumption based on their subgroup.

$$P_j = [\%\Delta\bar{p}_{j1} \quad \%\Delta\bar{p}_{j2} \quad \cdots \quad \%\Delta\bar{p}_{j12}]$$

For a given product k, the proportionate changes in participation (ρ_{jk}) and conditional consumption (c_{ijk}) where $e^{(p)}$ represents elasticities from the participation matrix and $e^{(c)}$ the conditional consumption matrix, are given by:

$$\%\Delta\rho_{jk} = \sum_{i=1}^{12} \%\,\Delta\bar{p}_{jk_i} e^{(p)}_{k_i,k}$$

$$\%\Delta c_{jk} = \sum_{i=1}^{12} \%\,\Delta\bar{p}_{jk_i} e^{(c)}_{k_i,k}$$

The base case elasticities used in the model were estimated using Living Costs and Food Survey (LCFS) data from 2006 - 2017[29]. Using a generalised Tobit regression model, two 12 by 12 elasticity matrices were produced - one for participation elasticities and one for conditional consumption elasticities. For sensitivity analyses, the model can use other elasticities instead, i.e. produced by other methods or comparing to other elasticity estimates in the literature.

Any alternative elasticity estimates can be used provided they are: (i) represented in the 12*12 product formulation and (ii) are input as a pair of participation and conditional consumption elasticities. Alternative elasticity matrices estimated using UK data which can be used by the TAX-sim model in sensitivity analyses include Meng et al.[30] for alcohol, and HMRC estimates by Sousa[31] (alcohol) and Czubeck and Johal[32] (tobacco).

5.4 Base Case and Sensitivity Analysis

One limitation to the use of elasticities in the TAX-sim model is that there is currently no capacity to investigate statistical uncertainty in the elasticity estimates. Each price elasticity has an associated standard error and confidence intervals reflecting the uncertainty in the statistical modelling which produced the estimates. As a single simulation takes approximately 12 hours to run, a probabilistic sensitivity analysis which can fully explore the impact of uncertainty in the estimates of the price elasticities is infeasible.

Sensitivity analysis which is routinely undertaken is a set of scenario analyses which address the structural uncertainty arising from different methods and data used to produce the estimates of price elasticities which exist in the literature. Structural sensitivity analysis is also undertaken to partially address the statistical uncertainty in the base case by switching on or off price elasticities which are not statistically significant.

In the present analysis, the price elasticity scenarios considered are:

- **Base case**. Pryce et al. 2023[29] elasticities using significant own and cross-price elasticities for tobacco and alcohol, with all non-significant price elasticities set equal to zero. See Table A1 and Table A2 for participation and conditional consumption elasticities respectively.
- **S1**. Pryce et al. 2023 elasticities using all elasticities including non-significant.
- **S2**. Base case elasticities excluding all cross-price elasticities between alcohol and tobacco.
- **S3**. Pryce et al. 2023 elasticities calibrated to alternative elasticities estimated by HMRC (see Section 9).

Table A1: Base case participation price elasticities of demand

	off beer	off cider	off wine	off spirits	off rtds	on beer	on cider	on wine	on spirits	on rtds	FM cigs	HRT
off beer	-0.25	0.00	0.00	-0.05	0.00	-0.03	0.00	0.00	-0.00	0.00	-0.05	-0.02
off cider	-0.03	-0.12	-0.02	-0.04	0.00	0.00	-0.01	0.00	0.00	0.00	0.00	0.00
off wine	0.00	0.00	-0.31	-0.03	0.00	0.00	0.00	0.00	0.00	-0.00	0.00	0.00
off spirits	0.00	0.00	0.00	-0.20	0.00	0.00	0.00	0.00	0.00	0.00	0.00	0.00
off rtds	0.00	0.00	-0.03	-0.03	-0.03	0.00	0.00	0.00	0.00	0.00	0.00	0.01
on beer	0.00	0.00	0.05	-0.01	0.00	-0.29	-0.02	0.09	0.00	0.00	-0.09	0.00
on cider	0.00	-0.02	0.00	-0.01	0.00	0.00	-0.09	0.00	0.00	0.00	0.00	0.01
on wine	-0.02	0.00	-0.07	-0.02	-0.01	0.00	0.00	-0.24	-0.01	0.00	0.00	0.00
on spirits	0.00	0.00	-0.01	-0.03	0.00	-0.12	-0.03	0.00	-0.18	0.00	0.00	-0.02
on rtds	0.00	0.00	0.00	-0.02	0.00	0.00	0.00	0.00	0.00	-0.01	0.00	0.00
FM cigs	0.04	-0.03	0.30	0.02	0.00	0.21	0.00	0.12	0.00	0.00	-0.17	0.00
HRT	0.00	0.00	0.00	-0.05	0.00	0.00	-0.01	0.00	0.00	0.00	0.00	-0.09

Table A2: Base case conditional consumption price elasticities of demand

	off beer	off cider	off wine	off spirits	off rtds	on beer	on cider	on wine	on spirits	on rtds	FM cigs	HRT
off beer	-1.20	0.00	0.00	-0.11	0.00	-0.12	0.00	0.00	-0.21	0.00	-0.16	-0.11
off cider	-0.07	-1.14	-0.13	-0.10	0.00	0.00	-0.33	0.00	0.00	0.00	0.00	0.00
off wine	0.00	0.00	-0.34	-0.06	0.00	0.00	0.00	0.06	0.00	0.22	0.00	0.00
off spirits	0.00	0.00	0.00	-0.22	0.00	0.00	0.00	0.00	0.00	0.00	0.00	0.00
off rtds	0.00	0.00	-0.11	0.00	-0.49	0.00	0.00	0.00	0.00	0.00	0.00	0.16
on beer	0.00	0.00	0.08	0.00	0.00	-0.80	-0.13	0.15	0.00	0.00	-0.18	0.00
on cider	0.00	-0.17	0.00	0.00	0.00	0.00	-0.34	0.00	0.00	0.00	0.00	0.31
on wine	0.07	0.00	-0.04	0.00	0.19	0.00	0.00	-0.39	0.06	0.00	0.00	0.00
on spirits	0.00	0.00	0.06	0.00	0.00	-0.18	-0.17	0.00	-0.78	0.00	0.00	0.14
on rtds	0.00	0.00	0.00	0.00	0.00	0.00	0.00	0.00	0.00	-0.14	0.00	0.00
FM cigs	-0.29	-1.02	0.40	0.36	0.00	0.45	0.00	0.40	0.00	0.00	-0.51	0.00
HRT	0.00	0.00	0.00	0.00	0.00	0.00	-0.43	0.00	0.00	0.00	0.00	-0.23

5.5 Updating consumption

Updating consumption involves first updating the participation of individuals in consumption of products, and then the amount consumed by individuals who remain consumers after the participation decision is made.

5.5.1 Adjusting participation

Firstly the number of consumers who will switch consumption is calculated on a product by product basis. The prevalence within each subgroup j of consumption of product k prior to the price change - $\rho_{jk,t-1}$ - is calculated and matched with the relative changes calculated from the participation elasticities - $\%\Delta\rho_{jk,t}$. The new prevalence following the price change is then:

$$\rho_{jk,t} = \rho_{jk,t-1}(1 + \%\Delta\rho_{jk,t})$$

The change in the number of consumers by subgroup, $N_j^{(c)}$, is then simply the size of the subgroup, N_j, multiplied by the change in prevalence:

$$\Delta N_j^{(c)} = N_j(\rho_{jk,t} - \rho_{jk,t-1})$$

If the number of consumers is falling, then individuals in the subgroup are chosen at random to become non-consumers and have their consumption adjusted to zero. If the number of consumers who will switch is greater than the number of current consumers then all consumers in the subgroup have their consumption switched to zero.

Conversely, if the number of consumers is rising, then non-consumers in the subgroup are chosen at random to become consumers. If the number of consumers who will switch is greater than the number of current non-consumers then all remaining non-consumers in the subgroup will become consumers. Non-consumers are chosen at random to become consumers and their consumption is set to the mean consumption of current consumers within their subgroup.

5.5.2 Adjusting conditional consumption

With participation adjusted, the level of consumption is next adjusted to reflect changes to the amount of product consumed conditional on the individual being a consumer. As with participation, the new consumption is calculated by combining the initial (individual level) consumption with the relative changes at subgroup level calculated from the conditional consumption elasticities.

$$c_{ijk,t} = c_{ijk,t-1}(1 + \%\Delta c_{jk,t})$$

Note that individual subgroups are determined partly by level of alcohol and tobacco consumption. These subgroups change dynamically such that if, for example, the combined policy effects of a duty change result in a drinkers' mean weekly consumption reducing from the level of a harmful drinker to that of a hazardous drinker, their drinker category is updated to reflect the change in consumption. This represents a change from the SAPM approach, in which an individual's drinker category is static and defined by drinking at baseline. *Comparisons of outcomes within drinker and/or smoking categories across different models should therefore be treated with caution*, as the composition of those groups will not be identical even given the same initial synthetic population.

6. Modelling tax policy to price

In the control and intervention arms of the model, changes to tax policy are modelled year-on-year. From the model index year to the policy effect year historical rates of excise duty and value-added tax are applied. As price distributions are expressed in real-terms values, adjusted for inflation by the Retail Price Index (RPI). All specific duty rates per unit are also expressed in real-terms, and so it is assumed that it is **real terms changes in duty** that have an impact on the real price and so on consumption.

Prior to the policy effect year, the control and intervention arms of the model follow an identical time series of historical duty rates. In the policy effect year and subsequent years the control arm assumes that alcohol duty is increased in line with inflation in nominal/cash terms (and so frozen in real terms). Tobacco duty is assumed to continue to increase in line with the duty escalator policy of RPI + 2%. This is assumed to be the case in all future years following the intervention.

In the intervention arm, the policy effect year is the year in which a policy change occurs e.g. a 10% increase in alcohol duty. Note that while most interventions modelled occur in a specific year, there may be policies which are phased in over a longer period, e.g. a specified increase in duty which is incrementally achieved over a number of years. As in the control arm, alcohol duty is assumed to increase in line with inflation after the policy effect year and tobacco duty continues to increase in line with the escalator (though in the treatment arm the escalator can be adjusted, e.g. increased from 2% to 4% above RPI, or abolished entirely).

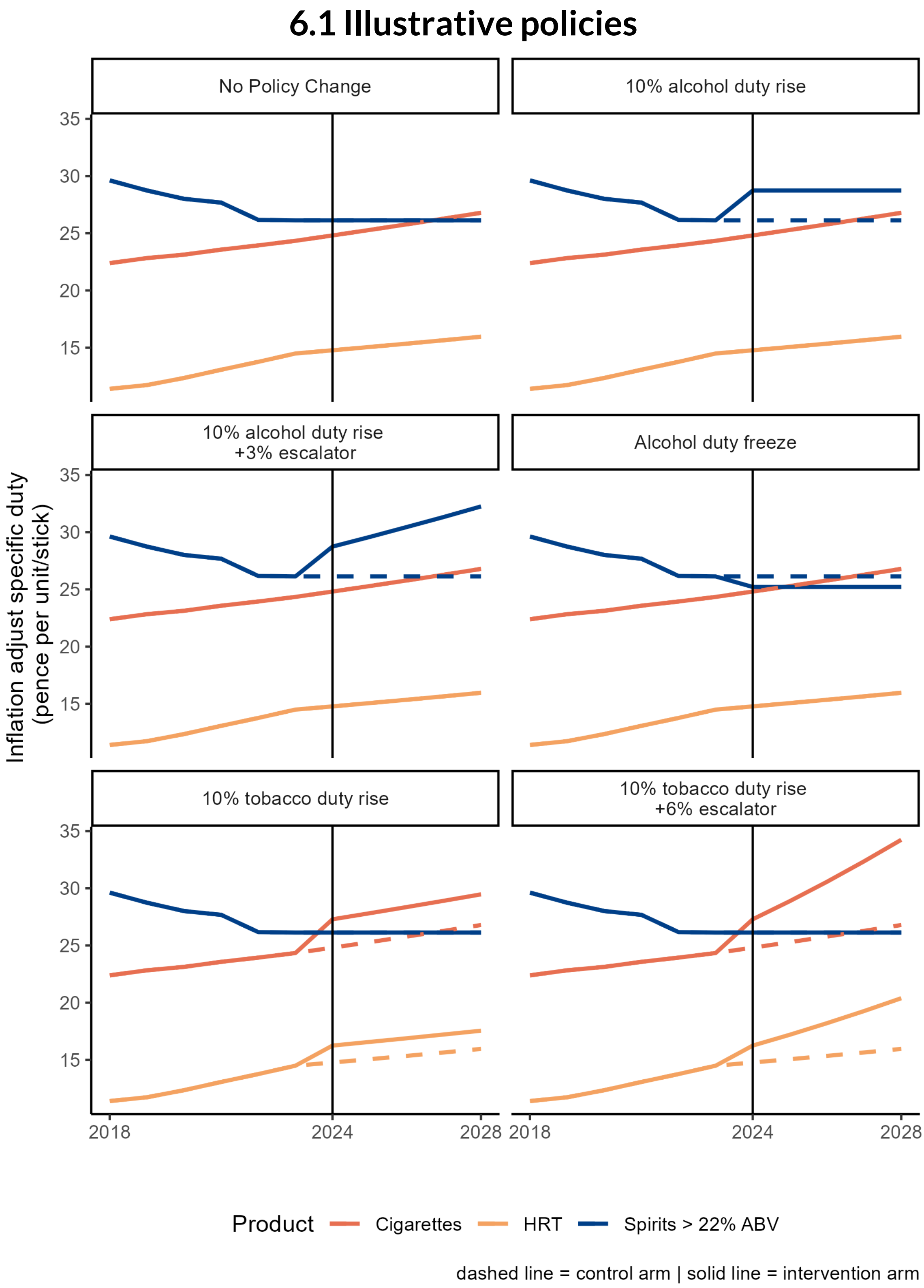

Figure 6.1: Illustrative tax policies - intervention vs control arm

Figure 6.1 presents some illustrative policy interventions and their comparisons with the business-as-usual control arm with a policy effect year in each case of 2024 - the year in which the control and intervention arms first diverge. The dashed lines represent the control arm and

solid lines represent the intervention arm and for illustrative purposes, factory-made cigarettes, hand-rolled tobacco, and spirits products stronger than 22% ABV.

The first policy represented is a "null" or no change model, to illustrate that the time series of duty rates for the control and intervention arms do not diverge in that case and would produce no policy effect. The next three policies adjust alcohol duty. Note that the control arm in each case is a horizontal line, representing the base case that duty is assumed to be held constant in real terms from the policy effect year onwards. This was not the case prior to 2023, where a period of duty freezes (for most alcohol products) in cash-terms resulted in a gradual eroding of the real-terms duty.

In addition to one-off duty changes, other possible tax policies that can be implemented/adjusted in the model include adding/modifying an annual real-terms escalator, modifying the rate of value-added tax (VAT), introducing or modifying an ad-valorem tax (a percentage of the retail price, which is currently in place at 16.5% for factory-made cigarettes). Another possible policy is a minimum excise tax (MET) which is used in conjunction with specific duty and ad-valorem tax, and is discussed in more detail in Section 6.4.

6.2 Calculate expected price

For the simpler case of alcohol and roll-your-own tobacco, where there is only duty and VAT is paid on top of this, the price per unit is expressed in terms of VAT, specific duty, and residual net revenue to retailers. In the case of factory-made cigarettes there is also an ad-valorem component. Price per unit of transaction l for product k is expressed as:

$$p_{kl} = \frac{VAT}{1 + VAT} p_{kl} + D_k + NR_{kl} + AVT_k p_{kl} \qquad (6.1)$$

Equation 6.1 decomposes the retail price into the tax components, with net revenue (NR) to retailers calculated as a residual. Rearranging into an expression for calculating retail price from the net revenue, specific duty per unit (D), the ad-valorem tax rate (AVT), and the rate of value-added tax (VAT) yields Equation 6.2.

The first step in applying a tax policy change is to calculate the *expected price*, p^{EXP}, which results from a policy which changes any of the components D, AVT, or VAT. This is the price which would be set, with retailer net revenue held constant, under the new tax regime. It is calculated by substituting values for the tax parameters into Equation 6.2 and holding net revenue constant at its level prior to the policy change.

$$p_{kl} = \frac{NR + D}{1 - \frac{VAT}{1 + VAT} - AVT_k} \qquad (6.2)$$

The expected price calculated here assumes that the supply side of the alcohol/tobacco markets do not respond to changes in the levels of taxation. Assuming the new observed price to be the expected price calculated here does not allow for under-shifting (absorbing some of the tax increase) or over-shifting (increasing the price by more than needed to simply cover for increased tax burden). Expected prices are therefore further adjusted for tax passthrough, to obtain a new observed price following the tax policy change which reflects industry/retailer response to the tax changes.

6.2.0.1 Apply tax passthrough

Tax passthrough in the model is based on empirical estimates of industry behaviour. To estimate passthrough coefficients, quantile regressions are estimated at the mid-point of each

decile of the observed price distribution, with observed price as the outcome variable and expected price as the independent variable. The tax passthrough coefficients are used to calculate the new observed price by applying Equation 6.3

$$p_{kl}^{*(\theta)} = p_{kl} + (\hat{p}_{kl}^{EXP} - p_{kl})\beta^{(\theta)} \qquad (6.3)$$

The observed new price - P - for transaction l of product k is obtained by adding the pre-policy price p_{kl} to expected change in price - $\hat{p}_{k,l}^{EXP} - p_{kl}$ - multiplied by $\beta^{(\theta)}$, the passthrough coefficient for the decile of the overall price distribution in which transaction l falls. $\theta \in (1,2,\ldots,10)$ indexes the price distribution decile for product k in which transaction l is located.

The model allows for flexible specification of different passthrough coefficients, including an option for 100% passthrough with no over- or under-shifting behaviours. Figure 6.2 illustrates the base case coefficients used in the model, representing the most up-to-date empirical estimates for off-trade[24] alcohol, on-trade alcohol[25], and tobacco[26].

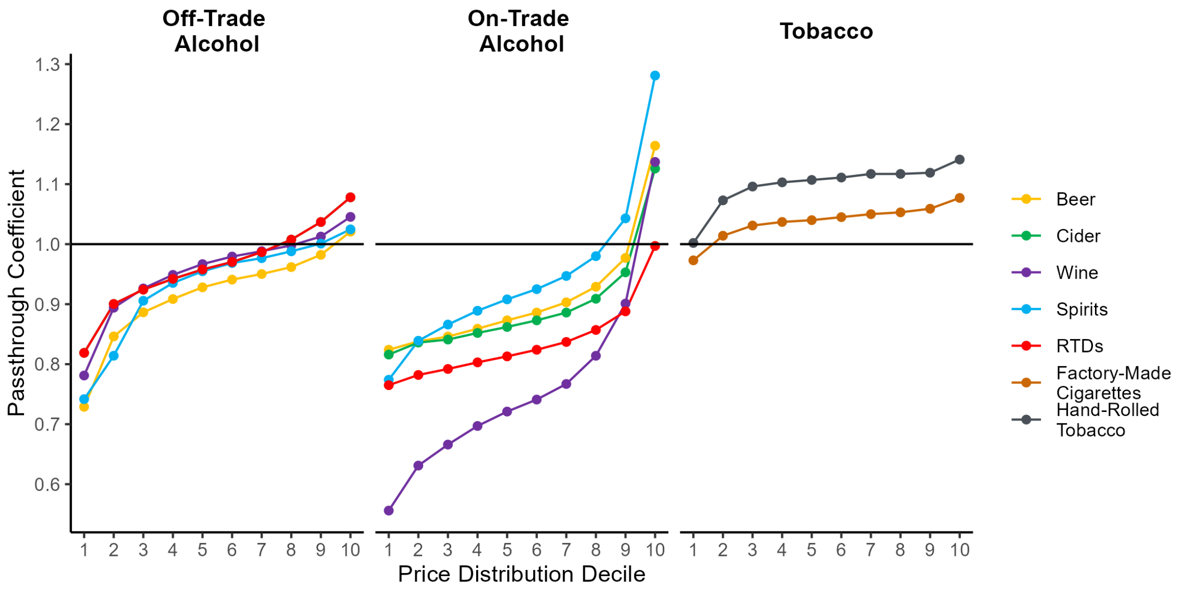

Figure 6.2: Base case tax passthrough coefficients for alcohol and tobacco products

6.3 Calculating the intervention effect

6.3.1 Average price change faced by consumers

The application of the passthrough coefficients to the expected price yields a set of price distributions adjusted for the changing tax structure and incorporating industry price response (which is modeled as the adjustments to tax passthrough above). The average price faced by a subgroup j for product k is simply the average of all price points in the price distribution weighted by the proportion of consumption occurring at each price point, as in Equation 6.4.

$$\bar{p}_{jk}^{*} = \sum_{l=1}^{50} (p_{kl}^{*} \times \delta_{jkl}) \qquad (6.4)$$

Applying this calculation to the updated price distribution and the price distribution from the previous year gives a pre and post-policy average price faced by each subgroup for each product, from which we can calculate the proportionate change in price at the subgroup level as in Equation 6.5.

$$\Delta\bar{p}_{jk,t} = \frac{\bar{p}^{*}_{jk,t} - \bar{p}^{*}_{jk,t-1}}{\bar{p}^{*}_{jk,t-1}} \qquad (6.5)$$

6.3.2 Calculation of net treatment effects

A key aspect to the price modelling not so far discussed is the calculation of net effects to avoid double counting in the calculation of the overall effect of a policy change in a given year. This double counting can occur as a result of the simulation conducting a forecast of consumption based on previous trends in consumption, which results in projected future trends in consumption that might implicitly assume the continuation of previous trends in pricing policy (e.g. that the tobacco duty escalator will continue to drive falls in tobacco consumption). Thus, the projected trends in the business-as-usual arm of the model reflect, in part, the effect of duty escalators in place for tobacco duties in the years used to estimate the likely trajectory of the future trend. Double counting occurs if the effect of the escalator impacts on consumption in the model both indirectly through the ongoing trends and directly through increases in taxation.

To accurately model the effect of a duty change in a given year, consumption is adjusted for the incremental difference between the new tax situation and the previous situation as described above. As the impact of the tobacco duty escalator is already accounted for in the underlying trends in tobacco consumption, the impact of the escalator must be subtracted from the policy effect of the tax change, to calculate a “net treatment” effect. This net treatment effect approach is applied in both the control / business-as-usual and the intervention arm(s) of the model.

To obtain an accurate treatment effect, both the control and intervention arm of the model is accompanied by the estimation of a policy effect from an additional counterfactual treatment arm. This counterfactual isolates the effect of any duty escalators by applying **any ongoing escalators to the tax regime of the previous year**. Calculating a change in the price distribution within this counterfactual arm calculates the change in price which is attributable to the escalator. This change in price in the counterfactual arm can then be subtracted from the total change in price observed in the main arm to leave only the impact of duty changes which are over and above the escalator.

This approach is formalised by Equation 6.6, which calculates the **changes in prices which will be attributed to the policy effect**. $\bar{p}^{*M}_{jk,t}$ refers to the average price in the main (M) arm and $\bar{p}^{*CF}_{jk,t}$ to the average price in the counterfactual (CF) treatment arm. The proportionate change in mean price is calculated as the change in price in the treatment arm net of the change in price in the counterfactual treatment arm as a proportion of the mean price in the previous year.

$$\Delta\bar{p}_{jk,t} = \frac{\left(\bar{p}^{*M}_{jk,t} - \bar{p}^{*}_{jk,t-1}\right) - \left(\bar{p}^{*CF}_{jk,t} - \bar{p}^{*}_{jk,t-1}\right)}{\bar{p}^{*}_{jk,t-1}} = \frac{\left(\bar{p}^{*M}_{jk,t}\right) - \left(\bar{p}^{*CF}_{jk,t}\right)}{\bar{p}^{*}_{jk,t-1}} \qquad (6.6)$$

6.3.3 Counterfactual adjustment

Note the behaviour of the counterfactual adjustment under the following scenarios:

1. *The product is not subject to an ongoing tax escalator.* In this case the price distributions in the counterfactual treatment arm at time t will be identical to the previous years price distribution i.e

$$\bar{p}^{*CF}_{jk,t} = \bar{p}^{*}_{jk,t-1}$$

and Equation 6.6 reduces to Equation 6.5, and all of the changes in prices that year represents a policy effect.

2. *The treatment arm in year t* only applies the ongoing escalator and no other tax changes. In this case the price distributions in the treatment and counterfactual treatment arms will be identical i.e.

$$\bar{p}^{*T}_{jk,t} = \bar{p}^{*CF}_{jk,t}$$

and the resulting change in mean price will be zero. Consequently none of the changes in prices that year are attributed to a policy effect.

3. *The treatment arm in year t* adjusts the ongoing escalator. In this case, e.g. the ongoing 2% escalator is increased to 3%, the price distributions will differ between the treatment and counterfactual treatment arms. The effect of the additional 1% will be attributed as a policy effect while the effect of the remaining 2% is captured in the underlying dynamics.

Note that the price distributions of the main arm reflect the reality of the price distributions that consumers will face in period $t + 1$, and so these price distributions are passed to the next year of the simulation as the price distribution input. The *counterfactual arm is used only to decompose the extent to which the change in the price distribution in period t is attributable to tax policy changes in that year rather than already being captured in the underlying dynamics of the model*. In scenario 2 above, although the change in price is calculated as zero *for the purpose of attributing treatment effects*, the presence of an escalator will still result in an updating of the price distributions which are passed on to the next year of the simulation.

6.4 Minimum excise tax

An additional form of excise duty, which is currently relevant in the UK in the case of tobacco but not alcohol, is a minimum excise tax. A minimum excise tax (MET) is a minimum total excise duty (which does not include VAT) that must be paid for a product. It sets a threshold level of duty that must be paid, and if the sum of specific duty and ad-valorem tax does not reach this threshold then the difference is paid in additional duty to satisfy the MET. Such a policy is applied to products with both a specific duty and ad-valorem tax. If there is no ad-valorem tax in place, then a MET is equivalent to simply setting a second specific duty rate, and the larger of the two values would apply. For this reason, MET is currently only in place for factory-made cigarettes - the only product in the model which is currently subject to both specific and ad-valorem duty.

The higher the price of a product, the more is paid in ad-valorem duty and the less likely a MET threshold is to be binding. The function of a MET is therefore to impose additional upward pressure on the prices of particularly cheap products. If price were set at some level, p^{MET}, such that the total of specific duty and ad-valorem duty paid equalled the MET threshold, retailers would have no incentive to lower the price further as the total tax payable would remain unchanged therefore leaving a smaller revenue margin. p^{MET} is the price at which no reduction in excise tax per unit can be achieved by lowering price further.

While a lower revenue margin (that the existence of a MET produces) might in theory be compensated for by a larger sales volume that would result from a lower price, this is unlikely to be the case in practice. The price elasticities for tobacco are generally much smaller than 1. The model default own-price elasticities for conditional consumption are -0.51 and -0.23

respectively for factory-made cigarettes and hand-rolled cigarettes. This means that any increases in consumption would be proportionately less than any price decrease. There is, consequently, no economic incentive for tobacco producers to lower the retail price below the level at which the MET threshold would be binding.

The TAX-sim model deals with a MET by assuming the MET threshold acts as an effective minimum unit price, given the previous discussion on the lack of incentive to reduce price. The core assumption of the model is that ***retailers set prices such that the MET threshold is always reached by the total excise duty paid at the retail price***. There is ***no price charged to consumers that would require additional tax to be paid on top of the specific duty and ad-valorem duty to reach the MET threshold***.

For a given MET threshold, the model calculates the corresponding minimum price at which the affected product will be sold. The approach to the MET is to determine the value p^{MET} which satisfies equation Equation 6.7 i.e. the price at which total excise duty paid is equal to the MET threshold:

$$MET_k^* = D_k^* + AVT_k^* p^{MET} \qquad (6.7)$$

The value of p^{MET} which satisfies Equation 6.7 is:

$$p^{MET} = \frac{MET_k^* - D_k^*}{AVT_k^*} \qquad (6.8)$$

p^{MET} then functions as a minimum unit price in the model. If the retail price falls below this, the amount of excise tax paid per unit remains constant and reduces the retail revenue per unit, removing the incentive to reduce the price further. If a MET is in place alongside a minimum unit price, the model will impose the larger of the two values to ensure the legal requirement of a minimum unit price and the economic incentives of a MET both hold.

This framework has so far been discussed with the implicit assumption that the minimum price dictated by the MET is equal to the value p^{MET}. This is a strong assumption, which is unlikely to be observed in reality, and is therefore relaxed in the implementation of the model using the methods described in Section 6.4.1.

6.4.1 Relaxing the binding MET assumption

As of TAX-sim version 2.5.0, the MET is handled more flexibly by allowing the minimum price dictated by the MET to be lower than p^{MET}. This is because the MET may not be fully binding in practice. Although in theory the MET threshold should be binding on industry there is evidence that cigarettes are being sold at a price per stick which is inconsistent with the assumption that the MET is fully binding. A search of the supermarket Asda website in January 2024 yielded a range of prices, the lowest of which was 56p per stick.

As of November 2023, the specific duty was £316.70 per 1,000 sticks and MET was £422.80 per 1,000 sticks, with an additional ad-valorem tax rate of 16.5% of the retail price. At a price of 56p per unit, the duty paid per stick is $£0.31670 + (16.5\% * £0.56) = £0.4091$. This is less than the MET per stick of £0.4228. This therefore violates the assumption in the model that the MET is fully binding and all prices increase such that the sum of specific and ad-valorem duty meets the MET threshold.

The minimum retail price per stick that can be charged if the MET is fully binding under this tax regime is $p^{MET} = \frac{(MET - D)}{AVT} = \frac{£0.4228 - £0.31670}{0.165} = £0.64$. This is the lowest price for factory-made

cigarettes in the model in 2024 when the assumption of a fully binding MET holds. The observed lowest price of £0.56 is therefore 87.5% of the modeled lowest price. Figure 6.3 illustrates the situation as of January 2024.

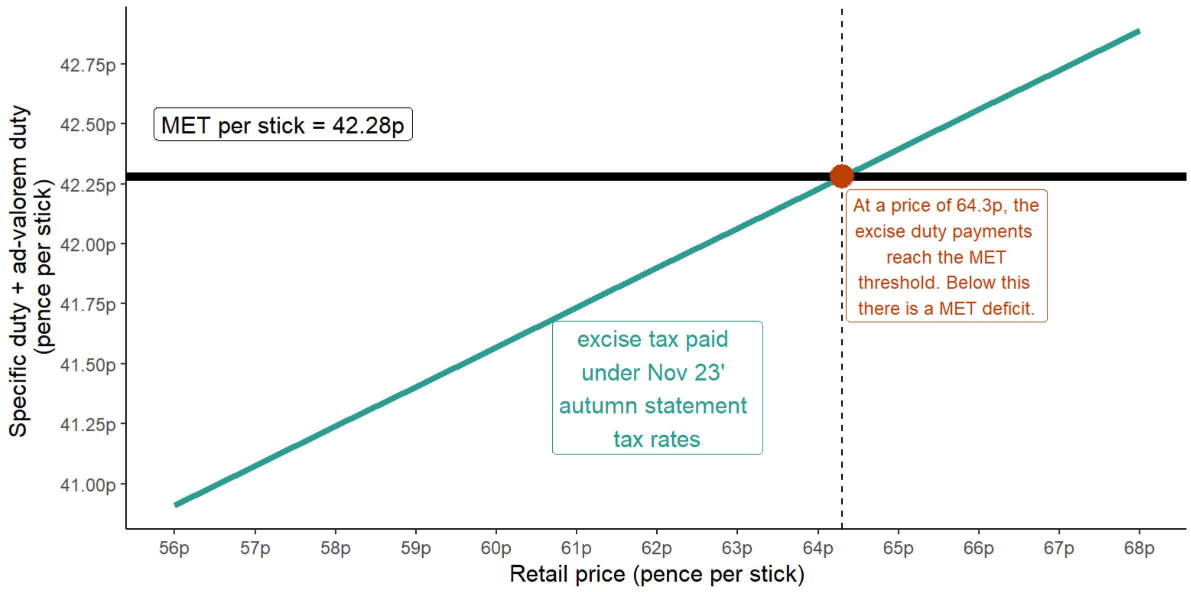

Figure 6.3: Effectiveness of the minimum excise tax for cigarettes in 2024

The assumption of a fully binding MET is relaxed by adding a parameter, α, to the model which is referred to as the *MET tolerance parameter*. This is a multiplier which is used to adjust the minimum price imposed by the model, to a value less than p^{MET}, allowing industry to set prices which lie below that implied by a fully binding MET. The minimum price set is $\alpha * p^{MET}$, where $0 \leq \alpha \leq 1$.

$$p^{minMET} = \alpha * p^{MET} = \alpha * \frac{MET_k^* - D_k^*}{AVT_k^*}$$

The value of α can vary over time in the simulation. The practical interpretation of the values of α are as follows:

- $\alpha = 0$. In this scenario the minimum price per stick for factory-made products as a result of the MET will always be zero ($p^{minMET} = 0$). This is an assumption that industry does not react at all to the presence of a MET in terms of price changes and simply pays the MET out of revenues from cheaper products.

- $\alpha = 1$. In this scenario, the minimum price per stick will be the price at which the sum of specific and ad-valorem duties meet the MET threshold ($p^{minMET} = p^{MET}$).

- $0 < \alpha < 1$. In this scenario, the minimum price per stick will be a price below the price at which the sum of specific and ad-valorem duties meet the MET threshold ($p^{minMET} < p^{MET}$). Depending on the value of α, this minimum will range between zero and p^{MET}.

The model allows for three specifications of the MET tolerance parameter:

1. **Fully binding MET**. The MET is always fully binding on the retail price. $\alpha = 1$ in all years.
2. **Fully ineffective MET**. The MET has no impact on price distributions at all. $\alpha = 0$ in all years.

3. **Eroding effectiveness**. At implementation $\alpha = 1$ in 2017. In 2024 $\alpha = 0.875$ based on the observable difference between the lowest price of cigarettes available and p^{MET}. Between 2017 and 2024, α decreases linearly from 1 to 0.875. After 2024, it remains at 0.875 indefinitely.

Option (3) is the base case used in the model.

7. Modelling wholesale price cap policy to price

A revenue cap, or price cap, is a policy which imposes a maximum price rather than a minimum price. The aim of such a policy is to restrict the revenues that industry can accrue from the sale of tobacco or alcohol, reducing profitability[33]. The price cap is imposed on the wholesale price (i.e. the price at which the manufacturer sells the product to the retailer), rather than the retail price. To mitigate any consequent fall in retail prices, the introduction of a price cap can be accompanied by a simultaneous rise in duty; the TAX-sim model can investigate the effects of different price cap thresholds combined with different levels of duty increase.

In the model, a price cap is interpreted as a cap on the net revenue NR component of the price equation (a proxy for the wholesale price), Equation 6.1. If a price cap, NR^{MAX} is set, then any net revenue values greater than the cap are reduced. Equation 6.2 is then used to re-calculate the retail price based on adjusted net revenue. As the impact of a price cap would be to reduce impacted retail prices below their equilibrium values, there may be an interaction between a price cap and any minimum unit price or minimum excise tax in place for the product. The retail price cannot fall below the threshold set by a pre-existing MUP or MET, and so in this scenario the price will only fall as far as the threshold, and any excess revenue above the cap that would accrue to retailers in this scenario is transferred to government as additional tax revenue.

$$p_{kl} = \frac{{NR_{kl}}^{*} + {D_k}^{*}}{1 - \frac{VAT}{1 + VAT} - AVT_k} \qquad (7.1)$$

When calibrating the duty rise required to keep the mean price for both tobacco products constant, we first adjust the wholesale price / net revenue to ${NR_{kl}}^{*}$, where ${NR_{kl}}^{*}$ is the wholesale price adjusted such that for product k at price point l in the price distribution, NR is reduced to the level of the price cap, if it initially exceeds the price cap.

The percentage change in duty required to keep the mean of the combined price distribution of both tobacco products constant is calculated by applying a uniform percentage increase to D in equation 7.1 for all products (k), and price points (l). We then solve numerically for the percentage increase in duty that produces the value ${D_k}^{*}$ that keeps the mean price constant for the given price cap.

The core assumption is that manufacturers respond to the price cap and duty increase primarily through price pass-through, consistent with observed responses to previous UK excise reforms. Retail prices are assumed to adjust mechanically to the cap and duty changes, without endogenous changes in product characteristics, marketing activity, or supply chains. Several alternative strategic responses are already constrained in the UK context.

Standardised packaging and minimum pack size regulations limit scope for pack size manipulation. Excise duties are levied on a per-stick (factory-made cigarettes) or per-gram (hand-rolling tobacco) basis, restricting opportunities for reformulation or blend-based tax avoidance, particularly given existing flavour restrictions. Tobacco advertising and promotion are comprehensively prohibited, leaving little scope for increased promotional activity. The wholesale price cap applies uniformly at the per-unit level, constraining the introduction of new low-price product lines.

8. Calculating economic outcomes

8.1 Calculating total spending, tax receipts, and industry revenue

Total spending and the division of total spending into tax revenues and retail/industry revenues are calculated by matching individual level consumption data to subgroup-level mean prices and tax payment data saved from the TAX-sim simulation run. Mean price, consumption, duty, and VAT are then calculated, stratified by product, year, and arm of the model. This calculation is weighted by the number of individuals in the total population each individual in the synthetic population represents. The calculation can additionally be stratified by any subgroup defined by variables in the consumption data e.g. by Index of Multiple Deprivation quintile.

Total consumption is calculated by multiplying the weighted mean of consumption by the total number of people in the population. Prior to this calculation, all consumption are adjusted to weekly figures (multiplying daily consumption of cigarettes and hand-rolled tobacco by 7). The total spending is then calculated as this total consumption figure multiplied by the mean price. Total VAT and total duty are similarly calculated as their respective subgroup-level means multiplied by total consumption in that subgroup. Total industry/retail revenue is then calculated as a residual - total expenditure minus total tax receipts. In addition to a mean price figure, a mean “basic price” figure is calculated, which is the retail price minus the tax components (equivalent to the net retail/industry revenue per unit of consumption). The weekly spending, tax, and revenue figures are then annualised by multiplying through by 52 weeks per year.

These calculations are performed for each of the 12 products in the model. The number of products can be aggregated. The two tobacco products can be left separated, or combined into an all-tobacco figure. There are a number of ways the alcohol figures can be aggregated over products; an all-alcohol figure similar to tobacco, a 2-product categorisation which aggregates all figures by on-trade or off-trade channel of sale, and a 5-product categorisation where figures are produced for beer, cider, wine, spirits, and RTDs separately and aggregated over channel of sale. Aggregation is performed by summing up the total tax, revenue, and spending figures, and as a mean weighted by consumption in the case of prices.

8.2 Upshifting

Upshifting is performed to account for under-reporting of alcohol consumption/spending in survey data. In the default configuration of the TAX-sim model, the model projects alcohol consumption and estimates the associated health outcomes based on **reported** consumption

(i.e. consumption that might be subject to under-reporting). However, in that default configuration, the estimates of total spending on alcohol, tax receipts, and industry revenues from alcohol sales have an adjustment applied to them to avoid underestimates in these outcomes due to the under-reporting of alcohol consumption. Thus, in the default model configuration, adjusted consumption figures are used to calculate more accurate figures for the economic outcomes, but the reporting of alcohol consumption and associated health outcomes is based on unadjusted consumption.

Upshifting of total spending on alcohol, tax receipts, and industry revenues from alcohol sales is performed by comparing the total duty receipts from alcohol estimated by the model with the actual total duty receipts reported by HM Revenue and Customs in a given reference year (by default, the reference year used is the year in which the model is initialised, but any year which is both included in the model and for which data from HMRC exists can be used). The data on actual duty receipts are obtained from the alcohol bulletin, currently available up to the 2021/22 financial year. In order to obtain figures relevant for the country within the UK to which the TAX-sim model is parameterised for, the total UK duty receipts from alcohol are disaggregated into country-specific estimates using an estimate of the country splits in duty receipts derived from an analysis of the Living Costs and Food Survey (LCFS) data.

Alcohol duty receipts are reported separately for beer, cider, wine, and spirits (alcopops are grouped with spirits) with no differentiation between on-trade and off-trade alcohol sales. Duty receipts estimated by the model are aggregated to match these four beverage categories and the upshift factor to be applied to the economic outcomes is calculated as the ratio of HMRC reported duty receipts to the un-adjusted duty receipts estimated by the model for each of the four beverage categories.

The four upshift factors are then used in all calculations of economic outputs from the model. Each of the 10 products in the model are assigned the upshift factor for the relevant 4-category disaggregation of the upshift factor. For example, the consumption of off-trade wine and on-trade wine are multiplied by the same factor. The upshifting to obtain adjusted economic outcomes is applied to the unadjusted estimates of individual-level consumption and this adjusted alcohol consumption is then multiplied by prices of products purchased to obtain the adjusted estimates of total spending on alcohol, tax receipts, and industry revenues from alcohol sales.

9. Sensitivity Analysis

For sensitivity analysis S3 we explore the impact on our results of using alternative, published price elasticities of demand for tobacco. Specifically, the price elasticities estimated by HMRC and used in their tobacco duty forecast model.

As the model is set up to use both participation and conditional consumption elasticities (presented for the base case in Table A3), we cannot simply replace the base case price elasticities with those estimated by HMRC. The HMRC elasticities are estimated using time series regression techniques and aggregated data on prices and clearances. We therefore do not have separate effects on participation and conditional consumption. To use the HMRC elasticities directly in the model we would have to make one of two assumptions:

- **Apply the elasticities to participation**. In this case we would obtain large participation effects in response to price changes and assume that those who remain consumers of

tobacco products after a price change do not change the amount of tobacco consumed at all.

- **Apply the elasticities to conditional consumption**. In this case no individuals quit consuming tobacco products in response to price changes, only change the amount they consume. Modelled impacts on prevalence would be zero.

Table A3: Base case price elasticities of demand for tobacco products

Change in price	Change in consumption	Factory-made cigarettes	Hand-rolling tobacco
**Participation**	**Factory-made cigarettes**	-0.17	0.00
	**Hand-rolling tobacco**	0.00	-0.09
**Conditional consumption**	**Factory-made cigarettes**	-0.51	0.00
	**Hand-rolling tobacco**	0.00	-0.23

These are both strong assumptions and are contrary to the evidence of Pryce and co-authors which shows statistically significant price elasticities for both participation and conditional consumption. Our method is to calibrate the base case price elasticities such that they produce the same impact as the HMRC on total consumption of the two tobacco products given the same proportionate change in price, using the following approach:

1. **Calculate HMRC-estimated price effects.** We apply a 10% reduction in price of factory-made cigarettes and a 10% increase in price of hand-rolling tobacco to the HMRC price elasticity matrix (Table A4), to calculate the effect on total consumption of factory-made cigarettes and hand-rolled tobacco. This results in a **12.1% increase for factory-made cigarettes** and a **13.0% reduction for hand-rolling tobacco**.

2. **Calculate base case price effects.** We then apply the same price change to the base case price elasticities (Table A3), to calculate the equivalent impacts for the base case. This results in a **5.3% increase for factory-made cigarettes** and a **2.4% reduction for hand-rolling tobacco**.

3. **Apply a global calibration factor.** We then apply a calibration to each of the four base case elasticities – factory-made cigarette and hand-rolling tobacco own-price elasticities, each for participation and conditional consumption. This global adjustment calibration factor is identical for each of the elasticities and is designed to align the estimated impact on consumption of factory-made cigarettes. A calibration factor of 2.29 applied to the base case elasticities results in a change in consumption of factory-made cigarettes of 12.1%, aligned with the HMRC elasticities.

4. **Apply a second calibration factor to hand-rolling tobacco.** Step 3 results in an estimated reduction in consumption of 5.4%, compared to the 13.0% reduction estimated using the HMRC elasticities. We therefore applied a second calibration factor

to the adjusted price elasticities from step 3, just to the two own-price elasticities for hand-rolling tobacco.

Table A4: HMRC price elasticities of demand for tobacco products

Change in price	Change in consumption	Factory-made cigarettes	Hand-rolling tobacco
	**Factory-made cigarettes**	-1.20	0.50
	**Hand-rolling tobacco**	0.01	-0.80

We use pre-intervention (2024) modelled baseline data on average amount consumed per consumer and prevalence of the two tobacco products. This approach allows us to use elasticities consistent with those estimated by HMRC in terms of effect sizes, while preserving the ability to model both participation and conditional consumption. The resulting calibrated elasticities for participation and conditional consumption are presented in Table A5, and we use these elasticities in the model for sensitivity analysis S3.

Table A5: Calibrated price elasticities of demand used in sensitivity analysis S3

Change in price	Change in consumption	Factory-made cigarettes	Hand-rolling tobacco
**Participation**	**Factory-made cigarettes**	-0.39	0.00
	**Hand-rolling tobacco**	0.00	-0.50
**Conditional consumption**	**Factory-made cigarettes**	-1.17	0.00
	**Hand-rolling tobacco**	0.00	-1.27

References

1 Morris D, Pryce R, Brennan A, Gillespie D, Wilson L, Angus C. Tobacco and alcohol tax and price intervention simulation model (TAX-sim): Full technical documentation. *Open Science Framework* 2023. DOI:10.17605/OSF.IO/KR23Z.

2 Gillespie D, Brennan A. Sheffield tobacco policy model (STPM): Full technical documentation. *Open Science Framework* 2023. DOI:10.17605/OSF.IO/FR7WN.

3 Gillespie D, Brennan A, Morris D, *et al.* The Sheffield alcohol policy model - new version coded in r (SAPM-r): Full technical documentation. *OSF* 2023. DOI:10.17605/OSF.IO/M37KT.

4 Coulson T, Tuljapurkar S. The dynamics of a quantitative trait in an age-structured population living in a variable environment. *The American Naturalist* 2008; **172**: 599–612.

5 Lee RD, Carter LR. Modeling and forecasting US mortality. *Journal of the American statistical association* 1992; **87**: 659–71.

6 Angus C, Henney M, Webster L, Gillespie D. Alcohol-attributable diseases and dose-response curves for the Sheffield alcohol policy model version 4.0. 2018. DOI:10.15131/shef.data.6819689.

7 Webster L, Angus C, Brennan A, Gillespie D. Smoking and the risks of adult diseases. School of Health; Related Research, University of Sheffield, 2018 https://figshare.com/articles/Smoking_and_the_risks_of_adult_diseases/7411451.

8 Prabhu A, Obi KO, Rubenstein JH. The synergistic effects of alcohol and tobacco consumption on the risk of esophageal squamous cell carcinoma: A meta-analysis. *Official journal of the American College of Gastroenterology| ACG* 2014; **109**: 822–7.

9 Hashibe M, Brennan P, Chuang S, *et al.* Interaction between tobacco and alcohol use and the risk of head and neck cancer: Pooled analysis in the international head and neck cancer epidemiology consortium. *Cancer Epidemiology Biomarkers & Prevention* 2009; **18**: 541–50.

10 Purshouse RC, Brennan A, Latimer N, Meng Y, Rafia R, Jackson R. Modelling to assess the effectiveness and cost-effectiveness of public health related strategies and interventions to reduce alcohol attributable harm in England using the Sheffield alcohol policy model version 2.0: Report to the NICE public health programme development group. University of Sheffield, 2009 http://www.nice.org.uk/guidance/index.jsp?action=download&o=45668.

11 Hill-McManus D, Angus CR, Meng Y, Holmes J, Brennan A, Meier P. Injury alcohol-attributable fractions: Methodological issues and developments. Health Economics; Decision Science, School of Health; Related Research (ScHARR), University of Sheffield, 2014 http://eprints.whiterose.ac.uk/97968/.

12 Hill-McManus D, Angus C, Meng Y, Holmes J, Brennan A, Meier PS. Estimation of usual occasion-based individual drinking patterns using diary survey data. *Drug and alcohol dependence* 2014; **134**: 136–43.

13 Watson PE, Watson ID, Batt RD. Prediction of blood alcohol concentrations in human subjects. Updating the Widmark equation. *Journal of studies on alcohol* 1981; **42**: 547–56.

14 Posey D, Mozayani A. The estimation of blood alcohol concentration: Widmark revisited. *Forensic science, medicine, and pathology* 2007; **3**: 33–9.

15 Cherpitel CJ, Ye Y, Bond J, Borges G, Monteiro M. Relative risk of injury from acute alcohol consumption: Modeling the dose–response relationship in emergency department data from 18 countries. *Addiction* 2015; **110**: 279–88.

16 Holmes J, Meier PS, Booth A, Guo Y, Brennan A. The temporal relationship between per capita alcohol consumption and harm: A systematic review of time lag specifications in aggregate time series analyses. *Drug and Alcohol Dependence* 2012; **123**: 7–14.

17 Kontis V, Mathers CD, Rehm J, *et al.* Contribution of six risk factors to achieving the 25× 25 non-communicable disease mortality reduction target: A modelling study. *The Lancet* 2014; **384**: 427–37.

18 Oza S, Thun MJ, Henley SJ, Lopez AD, Ezzati M. How many deaths are attributable to smoking in the united states? Comparison of methods for estimating smoking-attributable mortality when smoking prevalence changes. *Preventive medicine* 2011; **52**: 428–33.

19 Group DPPR. Reduction in the incidence of type 2 diabetes with lifestyle intervention or metformin. *New England journal of medicine* 2002; **346**: 393–403.

20 Mansournia MA, Altman DG. Population attributable fraction. *Bmj* 2018; **360**.

21 Rosen L. An intuitive approach to understanding the attributable fraction of disease due to a risk factor: The case of smoking. *International Journal of Environmental Research and Public Health* 2013; **10**: 2932–43.

22 Brennan A, Meier P, Purshouse R, *et al.* The Sheffield Alcohol Policy Model - A Mathematical Description. *Health Economics* 2014; **24**: 1368–88.

23 Gunning-Schepers L. The health benefits of prevention: A simulation approach. 1988.

24 Ally AK, Meng Y, Chakraborty R, *et al.* Alcohol tax pass-through across the product and price range: do retailers treat cheap alcohol differently? *Addiction* 2014; **109**: 1994–2002.

25 Wilson LB, Pryce R, Angus C, Hiscock R, Brennan A, Gillespie D. The effect of alcohol tax changes on retail prices: how do on-trade alcohol retailers pass through tax changes to consumers? *The European Journal of Health Economics* 2021; **22**: 381–92.

26 Wilson LB, Pryce R, Hiscock R, Angus C, Brennan A, Gillespie D. Quantile regression of tobacco tax pass-through in the UK 20132019. How have manufacturers passed through tax changes for different tobacco products? *Tobacco Control* 2020; **30**: e27–32.

27 Wagenaar AC, Salois MJ, Komro KA. Effects of beverage alcohol price and tax levels on drinking: a meta-analysis of 1003 estimates from 112 studies. *Addiction* 2009; **104**: 179–90.

28 Gallet CA, List JA. Cigarette demand: a meta-analysis of elasticities. *Health Economics* 2003; **12**: 821–35.

29 Pryce Robert, Wilson Luke, Gillespie D, Angus C, Morris D, Brennan A. Estimation of integrated price elasticities for alcohol and tobacco in the UK using the living costs and food survey 2006-2017. *Drug and Alcohol Review* 2023; **43**: 315–24.

30 Meng Y, Brennan A, Purshouse R, *et al.* Estimation of own and cross price elasticities of alcohol demand in the UKA pseudo-panel approach using the Living Costs and Food Survey 20012009. *Journal of Health Economics* 2014; **34**: 96–103.

31 Sousa J. Estimation of price elasticities of demand for alcohol in the united kingdom. *HMRC Working Papers* 2014.

32 Czubek M, Johal S. Estimation of price elasticities of demand for alcohol in the united kingdom. *HMRC Working Papers* 2010.

33 Branston JR, Gilmore AB. The case for Ofsmoke: the potential for price cap regulation of tobacco to raise £500 million per year in the UK. *Tobacco Control* 2013; **23**: 45–50.
