# Supplementary material for: Reducing tobacco supplier profits and pricing power: Modelling the impact of a tobacco price cap and tax increase on socioeconomic inequalities in England
Source: Soc Sci Med. 2026 Aug;402:119325. doi: 10.1016/j.socscimed.2026.119325 (PMC13249003; doi:10.1016/j.socscimed.2026.119325)
Supplement: Multimedia component 2 [file mmc2.pdf]

SUPPLEMENT B - SUPPLEMENTARY MATERIALS on RESULTS

Figure B1. The effect of implementing the wholesale price cap policies on the price distribution of tobacco.

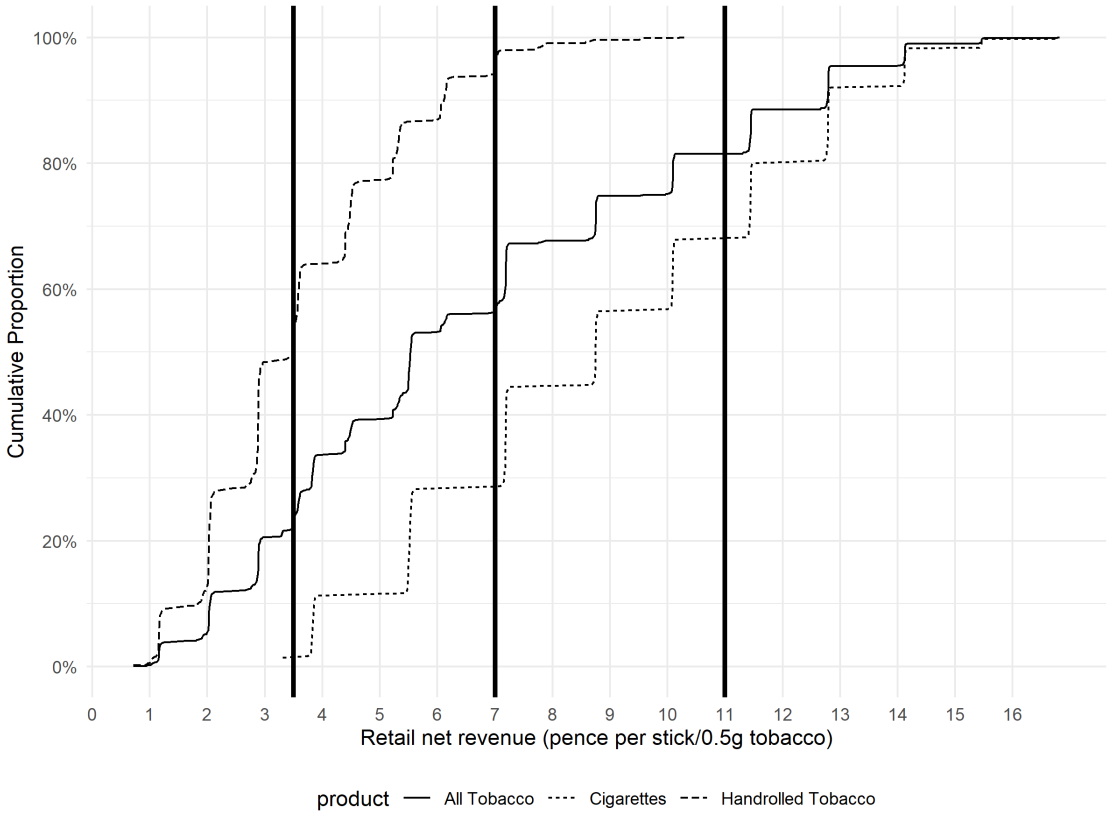

Figure B2. Compensating duty rise scenarios

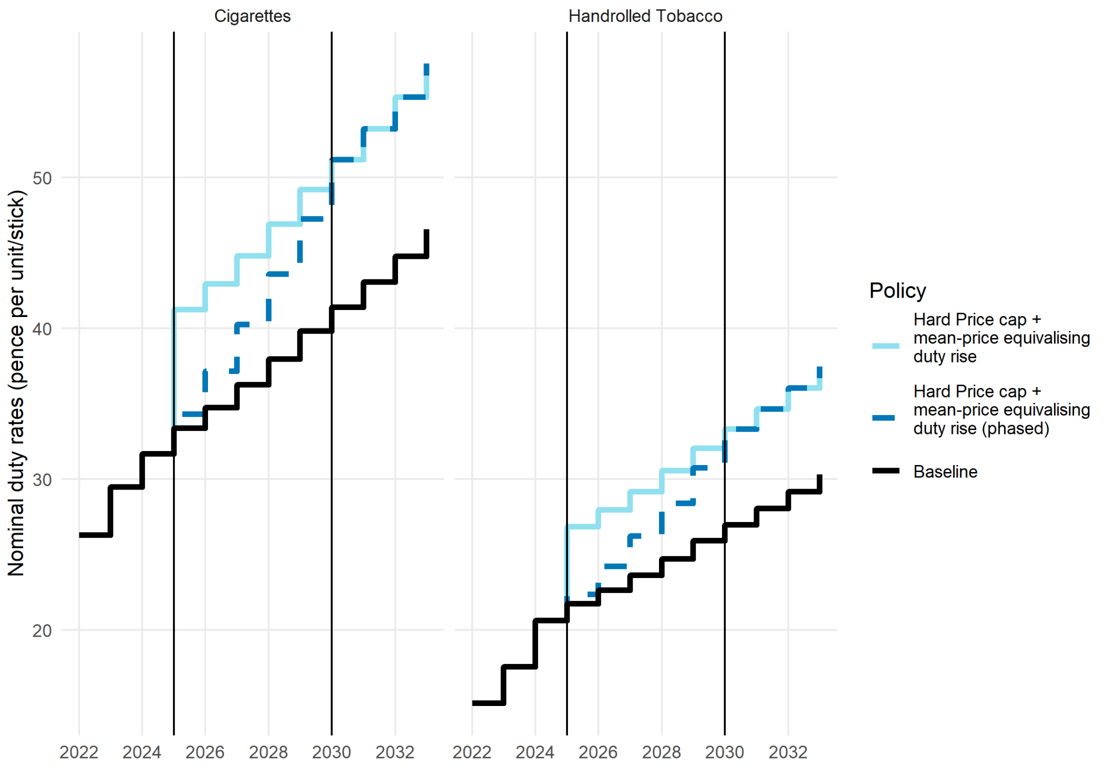

Table B1. Results of the sensitivity analysis that uses all cross-price elasticities including non-statistically significant ones (SA1). Impact of simultaneous price cap and compensating duty rise policies on smoking prevalence, spending, revenues, and health outcomes.

		Intervention arms (absolute difference from Control)					
	Control	Soft (immediate)	Moderate (immediate)	Hard (immediate)	Soft (phased)	Moderate (phased)	Hard (phased)
**Smoking prevalence in 2025 (% of population)**							
Population	***14.82***	-0.2	-0.38	-0.57	***0.02***	***-0.03***	***-0.09***
**Smoking prevalence in 2030 (% of population)**							
Population	***12.84***	-0.07	-0.11	-0.16	***-0.05***	***-0.13***	***-0.2***
IMDQ-1 (least)	***6.12***	-0.01	-0.03	-0.06	***-0.03***	***-0.05***	***-0.10***
IMDQ-2	***9.68***	-0.05	-0.07	-0.12	***-0.02***	***-0.09***	***-0.13***
IMDQ-3	***10.97***	-0.07	-0.09	-0.15	***-0.05***	***-0.11***	***-0.15***
IMDQ-4	***14.92***	-0.08	-0.13	-0.19	***-0.05***	***-0.15***	***-0.24***
IMDQ-5 (most)	***21.85***	-0.15	-0.22	-0.29	***-0.11***	***-0.22***	***-0.36***
**Mean tobacco consumption (cigarettes per person who smokes per week)**							
2025	***74.88***	-2.05	-3.92	-5.84	***0.32***	***-0.19***	***-0.91***
2030	***73.55***	-1.13	-2.15	-3.15	***-0.26***	***-1.46***	***-2.81***
**Mean spending on tobacco (£ per person who smokes per week)**							
2025	***39.66***	-0.94	-1.88	-2.89	***-0.22***	***-0.29***	***-0.38***
2030	***42.09***	-0.48	-0.99	-1.46	***-0.26***	***-0.69***	***-1.58***
**5-year Cumulative impact (2025-2029) on tobacco retail and tax revenues (£bn)**							
Retail revenue	***11.4***	-2.0	-4.5	-7.6	***-1.0***	***-1.9***	***-3.0***
Tobacco duty + VAT	***53.5***	0.2	0.9	2.2	***0.4***	***0.6***	***0.7***
**20-year Cumulative impact (2025-2044) on deaths, YLLs, admissions, and admissions costs**							
Deaths	***9,585,736***	522	-1,751	-1,625	***890***	***690***	***-706***
Years of life lost (YLL)	***141,974,540***	-28,636	-70,029	-83,432	***-18,566***	***-24,915***	***-63,473***
Hospital admissions	***40,666,745***	-5,251	-14,807	-15,690	***-5,844***	***-7,741***	***-13,089***
NHS admissions costs (£mn)	***74,812***	-9	-27	-28	***-11***	***-15***	***-24***

IMDQ = Index of Multiple Deprivation Quintile

The 'phased' scenarios achieve the same duty rates in 2030 as the corresponding 'immediate' scenarios but by increasing specific duty by the same percentage in each year over 2025-2029. The price cap for these scenarios is introduced gradually over 2025-2029

Table B2. Results of the sensitivity analysis that excludes statistically significant cross-price elasticities between tobacco and alcohol (SA2). Impact of simultaneous price cap and compensating duty rise policies on smoking prevalence, spending, revenues, and health outcomes.

		Intervention arms (absolute difference from Control)					
	Control	Soft (immediate)	Moderate (immediate)	Hard (immediate)	Soft (phased)	Moderate (phased)	Hard (phased)
**Smoking prevalence in 2025 (% of population)**							
Population	***14.89***	-0.1	-0.19	-0.29	***0.01***	***-0.01***	***-0.05***
**Smoking prevalence in 2030 (% of population)**							
Population	***12.95***	-0.02	-0.04	-0.08	***-0.01***	***-0.05***	***-0.09***
IMDQ-1 (least)	***6.14***	-0.01	-0.01	-0.03	***0.02***	***0.00***	***-0.03***
IMDQ-2	***9.76***	-0.03	-0.02	-0.05	***-0.02***	***-0.03***	***-0.07***
IMDQ-3	***11.06***	-0.02	-0.04	-0.06	***-0.01***	***-0.05***	***-0.06***
IMDQ-4	***15.07***	-0.03	-0.06	-0.10	***0.00***	***-0.06***	***-0.11***
IMDQ-5 (most)	***22.01***	-0.02	-0.09	-0.15	***-0.04***	***-0.08***	***-0.18***
**Mean tobacco consumption (cigarettes per person who smokes per week)**							
2025	***76.83***	-1.54	-2.89	-4.35	***0.26***	***-0.15***	***-0.72***
2030	***76.36***	-1.11	-1.43	-2.30	***-0.26***	***-1.05***	***-2.07***
**Mean spending on tobacco (£ per person who smokes per week)**							
2025	***40.19***	-0.57	-1.1	-1.81	***-0.22***	***-0.23***	***-0.23***
2030	***43.36***	-0.36	-0.1	-0.22	***-0.28***	***-0.37***	***-0.46***
**5-year Cumulative impact (2025-2029) on tobacco retail and tax revenues (£bn)**							
Retail revenue	***11.7***	-2.0	-4.4	-7.7	***-1.0***	***-1.9***	***-3.0***
Tobacco duty + VAT	***55.3***	0.6	2.4	4.5	***0.5***	***1.0***	***1.7***
**20-year Cumulative impact (2025-2044) on deaths, YLLs, admissions, and admissions costs**							
Deaths	***9,586,757***	42	-519	-863	***1,545***	***1,270***	***-447***
Years of life lost (YLL)	***141,965,583***	-3,370	-7,207	-23,370	***23,234***	***18,783***	***-7,870***
Hospital admissions	***40,647,614***	-439	-1,514	-3,485	***4,017***	***3,136***	***-852***
NHS admissions costs (£mn)	***74,770***	-1	-3	-6	***8***	***6***	***-1***

IMDQ = Index of Multiple Deprivation Quintile

The 'phased' scenarios achieve the same duty rates in 2030 as the corresponding 'immediate' scenarios but by increasing specific duty by the same percentage in each year over 2025-2029. The price cap for these scenarios is introduced gradually over 2025-2029

Table B3. Results of the sensitivity analysis that calibrates the base case price elasticities for tobacco to price elasticities estimated by HMRC (SA3). Impact of simultaneous price cap and compensating duty rise policies on smoking prevalence, spending, revenues, and health outcomes.

		Intervention arms (absolute difference from Control)					
	Control	Soft (immediate)	Moderate (immediate)	Hard (immediate)	Soft (phased)	Moderate (phased)	Hard (phased)
**Smoking prevalence in 2025 (% of population)**							
Population	***14.03***	-0.38	-0.73	-1.1	***0.03***	***-0.06***	***-0.17***
**Smoking prevalence in 2030 (% of population)**							
Population	***12.17***	-0.13	-0.23	-0.34	***-0.12***	***-0.27***	***-0.41***
IMDQ-1 (least)	***5.85***	-0.07	-0.10	-0.13	***-0.03***	***-0.10***	***-0.13***
IMDQ-2	***9.18***	-0.09	-0.16	-0.26	***-0.08***	***-0.20***	***-0.28***
IMDQ-3	***10.45***	-0.07	-0.14	-0.23	***-0.08***	***-0.18***	***-0.28***
IMDQ-4	***13.99***	-0.18	-0.36	-0.50	***-0.18***	***-0.42***	***-0.61***
IMDQ-5 (most)	***20.73***	-0.22	-0.38	-0.55	***-0.21***	***-0.44***	***-0.72***
**Mean tobacco consumption (cigarettes per person who smokes per week)**							
2025	***63.21***	-3.94	-7.42	-10.99	***0.64***	***-0.43***	***-1.83***
2030	***59.30***	-1.82	-3.54	-5.94	***-1.13***	***-2.86***	***-4.90***
**Mean spending on tobacco (£ per person who smokes per week)**							
2025	***36.52***	-1.73	-3.30	-4.92	***0.05***	***-0.30***	***-0.77***
2030	***36.67***	-0.42	-1.16	-2.03	***-0.61***	***-1.44***	***-2.24***
**5-year Cumulative impact (2025-2029) on tobacco retail and tax revenues (£bn)**							
Retail revenue	***9.8***	-2.1	-4.5	-7.2	***-1.0***	***-2.0***	***-3.1***
Tobacco duty + VAT	***45.7***	-1.1	-1.5	-1.7	***0.1***	***-0.7***	***-1.6***
**20-year Cumulative impact (2025-2044) on deaths, YLLs, admissions, and admissions costs**							
Deaths	***9,565,065***	-3,616	-3,600	-6,417	***-1,012***	***-2,507***	***-4,541***
Years of life lost (YLL)	***141,488,911***	-75,204	-102,541	-163,128	***-26,309***	***-61,174***	***-108,955***
Hospital admissions	***40,587,366***	-12,434	-16,123	-24,130	***-6,183***	***-11,780***	***-17,885***
NHS admissions costs (£mn)	***74,665***	-23	-29	-43	***-12***	***-22***	***-32***

IMDQ = Index of Multiple Deprivation Quintile

The 'phased' scenarios achieve the same duty rates in 2030 as the corresponding 'immediate' scenarios but by increasing specific duty by the same percentage in each year over 2025-2030. The price cap for these scenarios is introduced gradually over 2025-2030
